# Stroke network performance during the first COVID-19 pandemic stage: A meta-analysis based on stroke network models

**DOI:** 10.1177/17474930211041202

**Published:** 2021-08-28

**Authors:** Michele Romoli, Paolo Eusebi, Stefano Forlivesi, Mauro Gentile, Fabrizio Giammello, Laura Piccolo, David Giannandrea, Simone Vidale, Marco Longoni, Matteo Paolucci, Jessica Hsiao, Emily Sayles, Leonard LL Yeo, Espen Saxhaug Kristoffersen, Angel Chamorro, Liqun Jiao, Pooja Khatri, Georgios Tsivgoulis, Maurizio Paciaroni, Andrea Zini

**Affiliations:** 1 IRCCS Istituto delle Scienze Neurologiche di Bologna, Neurology and Metropolitan Stroke Center, “C.A. Pizzardi” Maggiore Hospital, Bologna, Italy; 2 Neurology and Stroke Unit, Department of Neuroscience, “Maurizio Bufalini” Hospital, Cesena, Italy; 3 Neurology Clinic, University of Perugia – S. Maria della Misericordia Hospital, Perugia, Italy; 4 Public Health Authority, Regione Umbria, Perugia, Italy; 5 International PhD in Translational Molecular Medicine and Surgery, Department of BIOMORF – University of Messina, Messina, Italy; 6 Neurologia e Stroke Unit, Ospedale di Gubbio e Gualdo Tadino, Perugia, Italy; 7 Neurology Unit, Rimini “Infermi” Hospital, AUSL Romagna, Rimini, Italy; 8 Department of Neurology, University of Cincinnati, USA; 9 Division of Neurology, Department of Medicine, National University Hospital, National University Health System, Singapore, Singapore; 10 Yong Loo Lin School of Medicine, National University of Singapore, Singapore, Singapore; 11 Department of Neurology, Akershus University Hospital, Lørenskog, Norway; 12 Department of General Practice, HELSAM, University of Oslo, Oslo, Norway; 13 Comprehensive Stroke Center, Department of Neuroscience, Hospital Clinic, University of Barcelona, Barcelona, Spain; 14 “August Pi i Sunyer” Biomedical Research Institute (IDIBAPS), Barcelona, Spain; 15 Department of Neurosurgery, Xuanwu Hospital, Beijing, China; 16 Department of Neurology, University of Tennessee Health Science Center, Memphis, USA; 17 Second Department of Neurology, “Attikon” University Hospital, School of Medicine, National and Kapodistrian University of Athens, Athens, Greece; 18 Neurology – Stroke Unit, Ospedale San Giuseppe, IRCCS MultiMedica, Milano, Italy

**Keywords:** Stroke, mothership, drip and ship, stroke network, COVID

## Abstract

**Background:**

The effect of the COVID pandemic on stroke network performance is unclear, particularly with consideration of drip&ship vs. mothership models.

**Aims:**

We systematically reviewed and meta-analyzed variations in stroke admissions, rate and timing of reperfusion treatments during the first wave COVID pandemic vs. the pre-pandemic timeframe depending on stroke network model adopted.

**Summary of findings:**

The systematic review followed registered protocol (PROSPERO-CRD42020211535), PRISMA and MOOSE guidelines. We searched MEDLINE, EMBASE, and CENTRAL until 9 October 2020 for studies reporting variations in ischemic stroke admissions, treatment rates, and timing in COVID (first wave) vs. control-period. Primary outcome was the weekly admission incidence rate ratio (IRR = admissions during COVID-period/admissions during control-period). Secondary outcomes were (i) changes in rate of reperfusion treatments and (ii) time metrics for pre- and in-hospital phase. Data were pooled using random-effects models, comparing mothership vs. drip&ship model. Overall, 29 studies were included in quantitative synthesis (n = 212,960). COVID-period was associated with a significant reduction in stroke admission rates (IRR = 0.69, 95%CI = 0.61–0.79), with higher relative presentation of large vessel occlusion (risk ratio (RR) = 1.62, 95% confidence interval (CI) = 1.24–2.12). Proportions of patients treated with endovascular treatment increased (RR = 1.14, 95%CI = 1.02–1.28). Intravenous thrombolysis decreased overall (IRR = 0.72, 95%CI = 0.54–0.96) but not in the mothership model (IRR = 0.81, 95%CI = 0.43–1.52). Onset-to-door time was longer for the drip&ship in COVID-period compared to the control-period (+32 min, 95%CI = 0–64). Door-to-scan was longer in COVID-period (+5 min, 95%CI = 2–7). Door-to-needle and door-to-groin were similar in COVID-period and control-period.

**Conclusions:**

Despite a 35% drop in stroke admissions during the first pandemic wave, proportions of patients receiving reperfusion and time-metrics were not inferior to control-period. Mothership preserved the weekly rate of intravenous thrombolysis and the onset-to-door timing to pre-pandemic standards.

## Introduction

Stroke care is based on fast rescue, rapid assessment, quick transportation according to local stroke network model and standardized management.^1,2^ Rapid definition of stroke syndromes and reperfusion of salvageable tissue is mandatory to increase chances of living in functional independence later in life.^1–3^

In December 2019, the emergence of the severe acute respiratory syndrome coronavirus 2 (SARS-CoV-2), in China, gradually evolved into a pandemic, with the death toll steadily increasing. The outbreak has forced national and local authorities to adapt emergency services to the need of the hour, and to impose global and unprecedented restrictions, from social distancing to national lockdown. The impact of such regulations on people perception of health problems and on their seeking of medical care has yet to be fully understood. Preliminary reports highlighted a potential contraction in presentation to the emergency department (ED) for acute time-dependent diseases, including acute ischemic stroke.^4–9^ However, large studies also reported higher rates of stroke admissions during the pandemic,^4,10^ potentially attributable to a higher risk of stroke with SARS-CoV-2 infection.^11,12^ Therefore, we lack conclusive data to quantify the variation in stroke admissions and time metrics of treatments provided.

The impact of the organization of the stroke network on the management of acute ischemic stroke under such unprecedented circumstances has been unexplored. Mothership model, based on direct admission to a comprehensive stroke center, and drip&ship (D&S) model, based on first assessment in spoke centers and transfer to a hub center, can critically impact time metrics.^2,13^ Therefore, the stroke network model might have consistently contributed to the variations in performance of the pre- and in-hospital service through the pandemic. As the pandemic still spreads, with countries still knee-deep in second waves, understanding how our stroke networks have adapted on a global scale seems crucial to program what to do in the near future, and advocate with health institutions for local needs.

Here, we provide a systematic review and meta-analysis of stroke admissions, rate, and timing of reperfusion treatments during the first phase of COVID-19 pandemic in comparison to a standard-of-care timeframe. We included stroke network organization as a variable to define the potential benefit of a chosen paradigm in such peculiar circumstances.

## Materials and methods

### Search strategy

The methods and guidelines of this study-level meta-analysis followed the PRISMA^14^ and MOOSE^15^ guidelines. The study protocol was registered with PROSPERO (CRD42020211535, principal investigator (PI) – M Romoli). We systematically searched Pubmed, EMBASE, and Cochrane CENTRAL for studies addressing ischemic stroke admissions, treatment, and time metrics across the COVID-19 pandemic (COVID-period) published from 2 June 2019 to 9 October 2020. Such timeframe was used to ensure a strict focus on the first phase of the pandemic, allowing for variations according to spread across countries. We used the following keywords in addition to Medical Subject Headings terms to build up the search protocol: *ischemic stroke, cerebrovascular disease, cerebrovascular accident, coronavirus, severe acute respiratory syndrome coronavirus 2, COVID, COVID19, COVID-19, novel coronavirus, nCoV, SARS-CoV2* (Supplementary Material). We used forward and backward citation tracking to improve the results.

### Eligibility criteria

We included all studies, prospective or retrospective, reporting original data on variations in stroke epidemiology, admissions, and stroke network performance over the COVID-period. We limited the studies to English language and excluded case reports, small case series (<30), conference proceedings/reviews. Studies not reporting data on consecutive stroke admissions or timing of rescue and treatment were excluded, as those including data for cumulative cerebrovascular diseases (e.g. hemorrhagic/ischemic stroke, stroke/TIA) or figure-only dissertation of undisclosed data. We also excluded duplicates of the same dataset and studies reporting stroke rates in patients diagnosed with SARS-CoV2. Four reviewers screened available literature (LP, MG, SF, FG) and selected studies according to pre-specified criteria; disagreement between reviewers was resolved by the corresponding author (MR).

### Data extraction and subgroup analysis

Four reviewers (LP, MG, SF, FG) independently evaluated the titles and abstracts of retrieved articles and screened for the full texts based on predefined criteria. Disagreements were resolved by the corresponding author. We collected data concerning design, stroke admissions, setting, population served, timeframes explored, baseline characteristics including National Institute of Health Stroke Scale (NIHSS) and treatment timing. We contacted authors of included studies reporting number of strokes in the two periods to improve results regarding stroke network organization, population served, and total number of stroke admissions and treatments provided. We reported the lack of data, when appropriate. When multiple distinct time periods were used as control-periods, whenever possible we selected data from the previous year to reduce the influence of seasonal variability; alternatively, summary and average measures from control-periods were used.^16^ Subgroup analysis was planned according to the stroke network model. Studies reporting data from multiple centers were considered according to the predominant network model whenever center-specific data were not available. For D&S and mothership definition, whenever a comprehensive stroke center received patients already treated with intravenous thrombolysis (IVT) to perform endovascular treatment (EVT), the paradigm was defined as D&S.

### Publication bias and risk of bias assessment

Publication bias was measured with the Egger’s regression test, and visualized in case of significant findings by funnel plots. Critical appraisal and methodological quality assessment was performed with the Newcastle-Ottawa scale (NOS), as previously validated and reported,^2,17^ and included assessment of selection of cohort explored, control cohort, length and adequacy of observation, and comparability of cohorts.^2,18^ We summarized the assessment as low, moderate, or high risk of bias.^17^

### Outcomes

The primary outcome was variation in weekly admission rate of stroke admissions during the COVID-period compared to the control-period. Study-dependent timeframes were used for all outcomes, as the pandemic evolved progressively but not contemporarily in all countries. The secondary outcomes included: (i) changes in weekly IVT and EVT rates, (ii) the proportion of IVT and EVT over those admitted, (ii) changes in time metrics for pre-hospital and in-hospital phase during COVID-period vs. control-period. Last-known-well/onset-to-door, door-to-needle, door-to-scan, door-to-groin, onset-to-needle, and door-to-reperfusion were extracted and calculated. Primary and secondary outcomes were also compared according to the stroke network model adopted (D&S vs. mothership).

### Statistical analysis

We pooled data from COVID-period vs. control-period according to original study definition. Outcome heterogeneity was evaluated with Cochrane’s Q-test and I^2^ statistic. An overall p-value of <.05 was considered statistically significant.

*Stroke admission and reperfusion rate ratio*: We calculated weekly incidence rates for admissions (IR) and reperfusion treatments (IR-IVT; IR-EVT) for all stroke centers with disclosed estimates of population served, using number of weeks of each timeframe to standardize result (IR = stroke/100,000/week). We pooled IR through meta-analysis of event count, deriving IR estimates for each timeframe (IR_COVID_, IR_control_). We then pooled the incidence rate ratio for stroke admissions (IRR = IR_COVID_/IR_control_),^19^ IVT and EVT through meta-analysis, displaying respective estimates for D&S and mothership models. Random-effects model (DerSimonian and Laird method) was preferred to account for heterogeneity in catchment area, design, and outcome assessment, as we previously reported.^2,18^ Combinatorial meta-analysis was implemented for the stroke admissions, IVT, and EVT IRR, and reported graphically.^2^ Meta-regression analysis was performed whenever trends of pooled estimates differed depending on network model, and if more than five studies were available overall.

*Reperfusion treatments*: We used meta-analysis of proportions to estimate the rate of IVT and EVT among all those admitted during each timeframe; our aim was to identify fluctuations in treatment rates rather than absolute reduction of treatments. Risk ratio (RR) was used to pool estimates, considering the control-period as baseline and the COVID-period as experimental. RR and respective 95% confidence interval (CI) were systematically calculated according to network model (D&S vs. mothership). We planned sensitivity analysis (i) excluding studies with unknown stroke network organization and (ii) including studies with prospective design only.

*Time measures*: Continuous variables, including stroke severity, as defined by NIHSS, and time metrics, were expressed as means and standard deviations. Onset/last-known-well-to-door, onset-to-needle, door-to-needle, door-to-scan, door-to-groin, and door-to-reperfusion were extracted. In cases where multiple time measures were available, time measures for IVT were selected for stroke-to-door and door-to-needle time, with time measures for door-to-groin were derived from EVT data. Mean and variance were calculated according to reported methodology whenever medians and IQR were available.^20^ We calculated mean difference (MD) in time (minutes) and NIHSS score (points) through meta-analysis of continuous outcome based on random-effect model, reporting MD and 95%CI. Meta-regression analysis was performed whenever differences in trend of pooled estimates emerged depending on network model, and if more than five studies were available overall. Sensitivity analysis involved: (i) analysis excluding studies with unknown stroke network organization and (ii) analysis including studies with prospective design only.

Data analysis was performed with R3.3.1.

## Results

### Literature review and characteristics of included studies

Of 461 records identified with systematic search, 110 fully assessed (Supplemental Figure I for PRISMA flow-chart, Supplemental Table I for excluded studies). Twenty-nine studies were included in qualitative synthesis (Table 1). Overall, data regarding 212,960 patients were collected from three continents (Europe, Asia, America). Mean length of the COVID-period was 7.2 ± 2.9 weeks vs. 11.2 ± 12.3 weeks for control-period, with the latter consisting of standard workflow data from 2019 in 16/29 studies (55.2%). COVID-period varied according to the spread of the pandemic, starting from January in studies performed in eastern countries,^21,22^ March in European countries,^4,23^ and up to May in western countries^24–26^ (Table 1). All studies focused on stroke admissions, none having appropriate catchment methods or pursuit to provide incidence data. Five studies had prospective data collection,^21,27–30^ while most of the studies were retrospective in nature. Four studies used data collected for the American Heart Association Get-with-the-guideline study.^10,24,31,32^ Two studies reported data from electronic-based registries, with aggregated data available only^16,25^; two studies were reperfusion registries^27,33^ and were therefore included for time metrics. Nine studies reported data on stroke networks organized according to a preferred mothership model^4,21–23,28,32,34–36^ (Table 1).

**Table 1.** Characteristics of the included studies

Reference	Year	Region (country)	Design	Setting	COVID start	COVID end	Stroke network model	Population served
Agarwal et al.^24^	2020	New York City (USA)	Retrospective^a^	Single center	1 Mar 2020	15 May 2020	Unknown	Unknown
Baracchini et al.^38^	2020	Veneto (Italy)	Retrospective	Single center	1 Feb 2020	31 Mar 2020	Drip&ship	Unknown
de Havenon et al.^16^	2020	USA	Retrospective	Multicenter	1 Feb 2020	31 Mar 2020	Unknown	Unknown
Diegoli et al.^28^	2020	Joinville (Brazil)	Prospective	Multicenter	17 Mar 2020	15 Apr 2020	Mothership	590,466
Esenwa et al.^39^	2020	New York City (USA)	Retrospective	Multicenter	1 Jan 2020	25 Feb 2020	Drip&ship	2,000,000
Hsiao et al.^40^	2020	Ohio, Kentucky, Indiana (USA)	Retrospective	Multicenter	9 Mar 2020	12 Apr 2020	Drip&ship	2,100,000
Huang et al.^37^	2020	Mayo Clinic (USA)	Retrospective	Multicenter	11 Mar 2020	09 Apr 2020	Drip&ship	Unknown
Jasne et al.^32^	2020	Connecticut (USA)	Retrospective^a^	Multicenter	1 Mar 2020	28 Apr 2020	Mothership^b^	Unknown
Kerleroux et al.^27 c^	2020	France	Prospective	Multicenter national level	15 Feb 2020	30 Mar 2020	Unknown	Unknown
Mehrpour et al.^41^	2020	Iran	Retrospective	Single center	15 Feb 2020	15 Apr 2020	Drip&ship	2,000,000
Montaner et al.^29^	2020	Seville (Spain)	Prospective	Multicenter	Unknown	Unknown	Drip&ship	2,450,000
Naccarato et al.^6^	2020	Trieste (Italy)	Retrospective	Single center	9 Mar 2020	09 Apr 2020	Drip&ship	373,803
Nguyen-Huynh et al.^10^	2020	Northern California (USA)	Retrospective^a^	Multicenter	15 Mar 2020	09 May 2020	Drip&ship	4,400,000
Onteddu et al.^25^	2020	USA	Retrospective	TriNetX platform	20 Jan 2020	16 May 2020	Unknown	Unknown
Paliwal et al.^34^	2020	Singapore	Retrospective	Single center	7 Feb 2020	30 Apr 2020	Mothership	1,300,000
Pandey et al.^19^	2020	Michigan and North-West Ohio (USA)	Retrospective	Multicenter	1 Mar 2020	31 Mar 2020	Unknown	Unknown
Perry et al.^35^	2020	North Central London (UK)	Retrospective	Single center	1 Apr 2020	11 May 2020	Mothership	1,270,000
Pop et al.^36^	2020	Alsace (France)	Retrospective	Multicenter	1 Mar 2020	31 Mar 2020	Mothership	1,900,000
Rudilosso et al.^30^	2020	Barcelona (Spain)	Prospective	Single center	1 Mar 2020	31 Mar 2020	Drip&ship^d^	2,740,000
Saxhaug Kristoffersen et al.^23^	2020	Akershus University Hospital (Norway)	Retrospective, observational	Single center	13 Mar 2020	30 Apr 2020	Mothership	570,000
Schirmer et al.^31^	2020	USA	Retrospective^a^	Multicenter	1 Mar 2020	28 Mar 2020	Unknown	Unknown
Siegler et al.^42^	2020	Cooper University Hospital (NJ, USA)	Retrospective	Single center	1 Mar 2020	15 Apr 2020	Drip&ship	2,500,000
Strasser et al.^26^	2020	Florida (USA)	Retrospective	Single center	1 Mar 2020	30 Apr 2020	Unknown	Unknown
Tejada Meza et al.^5^	2020	Aragon (Spain)	Retrospective	Multicenter	14 Mar 2020	19 Apr 2020	Drip&ship^e^	1,319,291
Tejada Meza et al.^43^	2020	Aragon (Spain)	Retrospective	Multicenter	09 Mar 2020	03 May 2020	Drip&ship^e^	11,500,000
Teo et al.^22^	2020	Hong Kong	Retrospective	Single center	23 Jan 2020	24 Mar 2020	Mothership	500,000
Yang et al.^21^	2020	Beijing (China)	Prospective	Single center	23 Jan 2020	07 Mar 2020	Mothership	Unknown
Zhao et al.^33^ ^c^	2020	China	Retrospective	Multicenter (China Registry)	01 Jan 2020	29 Feb 2020	Unknown	Unknown
Zini et al.^4^	2020	Bologna (Italy)	Retrospective	Single center	01 Mar 2020	30 Apr 2020	Mothership	1,100,000

a Get-with-the-guidelines AHA study (except Aragon, mothership).

b Network based on drip&ship, reported data only for comprehensive stroke center mothership admissions (own catchment area).

c Endovascular treatment registry.

d Except direct admission to comprehensive stroke center with mothership paradigm in limited central area.

e Except Aragon (mothership).

### Publication bias, risk of bias, and heterogeneity assessment

Funnel plots for studies reporting stroke admissions and reperfusion treatment ratio in the two periods explored are displayed in Supplemental Figure II. Egger’s regression test did not detect publication bias (p_Egger_ = .71, .27, and .96, respectively). Bias assessment demonstrated an overall good quality for included studies: 25/29 had low risk of bias (NOS score ≥ 7), 5 had low to moderate quality mainly in relation to selection and comparability bias (Supplemental Table II). Duration of observation for COVID-period was <5 weeks in nine studies.^6,19,28–31,35–37^ The study of lowest quality was excluded from quantitative analysis due to undisclosed data and ascertainment methods.^38^

**Figure 1.**
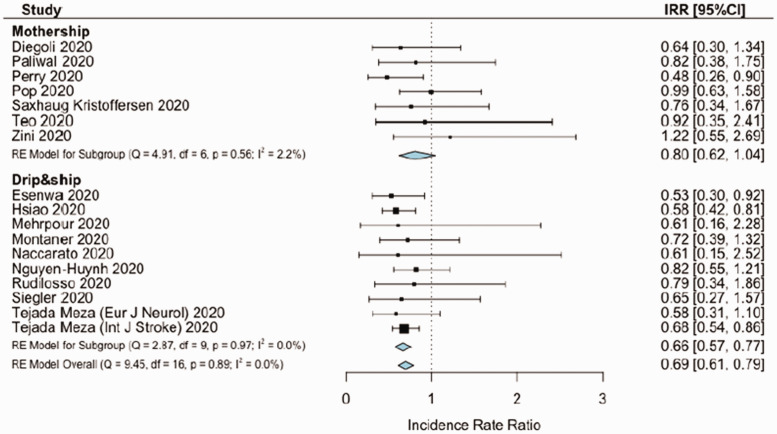
Meta-analysis of incidence rate ratio, comparing stroke admissions during COVID-period vs. control-period, depending on stroke network model.

**Figure 2.**
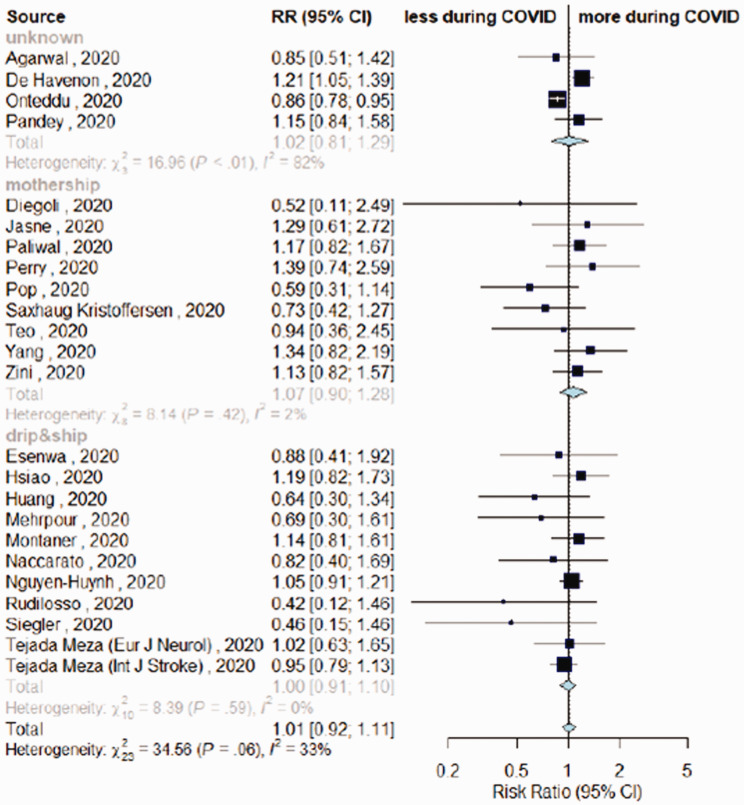
Differences in rate of intravenous thrombolysis (IVT) among admitted patients in COVID-19 vs. control-period, depending on stroke network model adopted.

**Figure 3.**
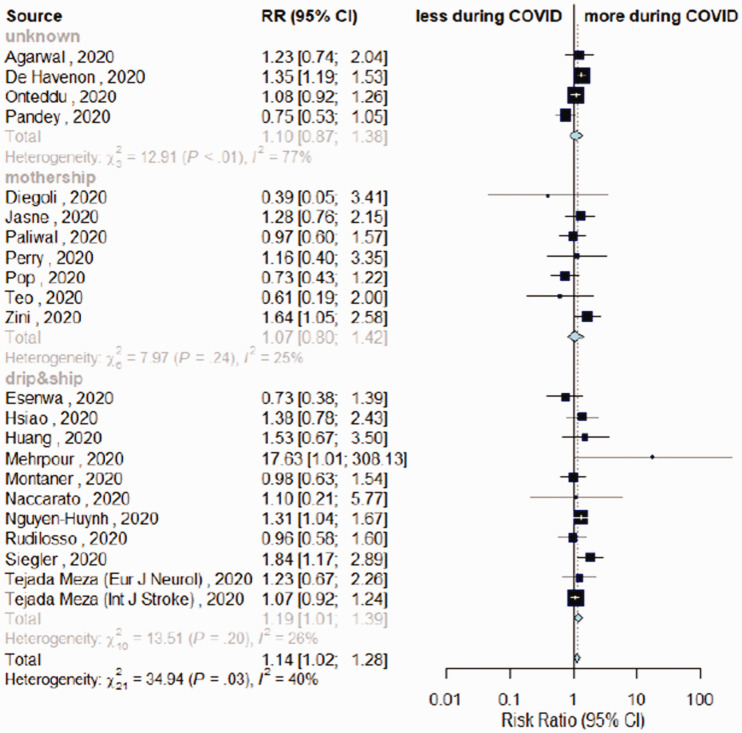
Differences in rate of endovascular thrombectomy (EVT) among patients admitted during COVID-19 vs. control-period, depending on stroke network model adopted.

**Figure 4.**
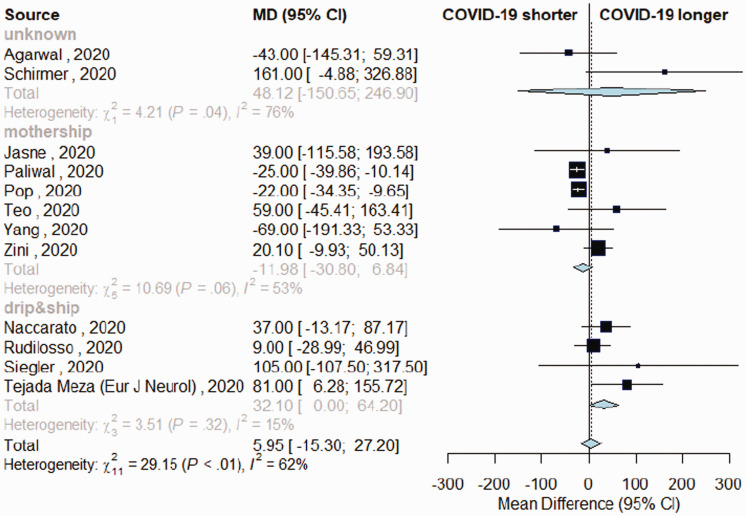
Variation in onset-to-door time in patients with acute ischemic stroke admitted during COVID-19 and control-period, depending on stroke network model adopted.

The overall estimates for the primary outcome, weekly IVT/EVT IRR and proportion of patients receiving IVT had low heterogeneity (I^2^ = 0%, p > .10; Figures 1 and 2, Supplemental Figure IV). Heterogeneity was mild for estimates of proportions of EVT among patients admitted (I^2^ = 40%, p_heterogeneity_ = .03, Figure 3) and onset-to-door time (I^2^ = 62%, p_heterogeneity_ < .01, Figure 4). In both cases, heterogeneity mainly derived from studies with unknown network organization (I^2^ > 70%, p_heterogeneity_ < .05, Figures 3 and 4), with estimates from D&S and mothership studies showing low heterogeneity (Figures 3 and 4, Supplemental Figure VIII). Door-to-groin had mild heterogeneity (I^2^ = 52%, p_heterogeneity_ = .04), with low heterogeneity in D&S stratum and mild heterogeneity in mothership stratum (I^2^ = 65%, p_heterogeneity_ = .02, Supplemental Figure XIII). Analysis of the remaining time metrics was conducted under low heterogeneity.

### Outcome of meta-analysis

#### Stroke admission and reperfusion treatment rate ratios

Seventeen studies reported data on stroke admissions during the pandemic and respective catchment areas, for a global served population of 38.6 million inhabitants across three continents (n = 38,613,560). COVID-period was associated with a significant reduction in stroke admission rate (IRR = 0.69, 95%CI = 0.61–0.79). COVID-period was associated with a slight trend in reduction of stroke admissions for mothership paradigm (IRR = 0.8, 95%CI = 0.61–1.04), while the reduction seemed significant for D&S model (IRR = 0.66, 95%CI = 0.57–0.77). Sensitivity combinatorial analysis did not highlight potential clusters or outliers (Supplemental Figure III). IVT weekly rates decreased during COVID-period (IRR = 0.72, 95%CI = 0.54–0.96). IVT weekly rates remained stable to pre-pandemic levels in mothership paradigm, while IVT weekly rates significantly decreased in D&S model (IRR-IVT = 0.7, 95%CI = 0.5–0.96) (Supplemental Figure IV). EVT rates were unchanged during COVID-period compared to control-period, independently from stroke network model (IRR-EVT = 0.79, 95%CI = 0.58–1.08) (Supplemental Figure IV). Mean NIHSS had a minor increase during COVID-period only in mothership studies (MD = 0.82, 95%CI = 0.05–1.58, Supplemental Figure V). Among people admitted with acute ischemic stroke in each timeframe, large vessel occlusions (LVO) were more frequent during COVID-period compared to control-period (RR = 1.62, 95%CI = 1.24–2.12, Supplemental Figure VI).

#### Reperfusion treatments

Twenty-four studies reported IVT among patients admitted during COVID- and control-period (n = 160,554).^4–6,10,16,19,21–25,28–30,32,34–37,39,40–43^ The proportion of people receiving IVT among those admitted for acute ischemic stroke was similar across timeframes (RR = 1.01, 95%CI = 0.92–1.11, Figure 2, Supplemental Figure VII).

Twenty-three studies reported EVT among patients admitted during the two timeframes.^4–6,10,16,19,21,22,24,25,28–30,32,34–37,39,40,41–43^ A higher proportion of people among those admitted received EVT during COVID-period compared to control-period (RR = 1.14, 95%CI = 1.02–1.28, Figure 3). A significant higher proportion of patients treated with EVT emerged during COVID-timeframe with D&S (RR = 1.19, 95%CI = 1.01–1.39), although not differing from mothership model (RR = 1.07, 95%CI = 0.80–1.42, p_meta-regression_ = 0.52). Sensitivity analysis removing studies with unknown network organization confirmed a marginal increase in EVT rates during COVID-period (RR = 1.12, 95%CI = 0.99–1.27; Supplemental Figure VIII).

#### Time metrics

Comparing onset-to-door time, no substantial differences emerged between periods (Figure 4). However, mothership and D&S models differed, with the latter associated with longer interval (MD +32 min, 95%CI = 0–64) and the former having non-significant shorter interval during COVID-period (MD –12 min, 95%CI = (-30)-(+7) minutes, Supplemental Figure IX). The mothership model had a significant impact on shortening onset-to-door time, as confirmed by meta-regression analysis (p = .03, Supplemental Table III). Door-to-scan time was longer during COVID-period in both models (MD = 5 min, 95%CI = 2–7, Supplemental Figure X). Door-to-needle, door-to-groin, onset-to-needle, and door-to-recanalization time were similar across timeframes (Supplemental Figures XI to XV), although for the latter data were not available from studies using D&S model.

## Discussion

This meta-analysis compared stroke network performance before and during the COVID-19 pandemic, revealing that, despite a substantial reduction in stroke admissions, the time metrics and the proportion of patients undergoing reperfusion treatments remained unchanged compared to pre-pandemic period. Stroke admissions dropped by 35%, a substantial reduction with slight impact from the stroke network model adopted, with the mothership model having marginally lower decline compared to D&S. At the same time, the proportion of people admitted with LVO increased, justifying the hypothesis that people with transient ischemic attack or very minor stroke might have avoided in-person consultation due to safety concerns.^4,7,40,44^ To this regard, considering that the proportion of patients undergoing reperfusion treatments and their time metrics were not reduced during the pandemic, stroke awareness campaigns seem to represent a priority to prompt people with minor stroke to seek medical attention. The only study that reported an increase in stroke admission rates during COVID-period was performed in Emilia-Romagna, a region advertising a stroke awareness campaign (https://salute.regione.emilia-romagna.it/campagne/ictus-vedo-riconosco-chiamo) immediately before the pandemic, providing a potential proof of concept.^4^

While proportions of patients treated with IVT among all those admitted with stroke were similar across timeframes, EVT increased during COVID-period. This should be put in context of a 60% relative increase in LVO presentation during the pandemic, potentially in relation to the avoidance of searching hospital/medical attention during the COVID-period in case of minor symptoms. Both mothership and D&S models had higher proportions of EVT, although mothership model associated with shorter onset-to-door time compared to D&S. The increase in rate of LVO and EVT might be attributable to several factors, first of all from the adoption of protocols in line with the DEFUSE3^45^ and DAWN^46^ trials. To this extent, the marginal increase in mean NIHSS during COVID-period supports a difference in diagnostic approach rather than only in stroke severity. At the same time, the potential vascular complications of SARS-CoV-2^11^ and the reduced compliance with antithrombotic medications during the pandemic^47,48^ might have further contributed to the higher risk of LVO. Regarding IVT variations, it might be useful to highlight that mothership preserved the rates of treatment during the COVID-period, as opposed to D&S, which faced a 30% reduction in weekly IVT rates. This result seems in line with a lower reduction in stroke admissions with mothership paradigm compared to D&S, which might have contributed to preserving the overall weekly IVT rates.

The longer onset-to-door time found in the D&S model has to be analyzed with caution. The stroke network model adopted depends on geography and logistics. Several hospitals hosting stroke spoke centers have been redeployed to COVID-hospitals, which might have impacted on time metrics of the stroke network, as well as on people safety concerns in accessing the ED.^49^ Mothership model might be more elastic compared to D&S given the fact that it usually deals with higher volumes, such as those happening during the pandemic, and might therefore more easily maintain the standards for timing of rescue and treatment.^2,4^ To this regard, we previously reported shorter timing for rescue and treatment after transitioning from a D&S to a mothership model during the pandemic,^50^ an option that might therefore be taken into account during the second wave. In this meta-analysis, the door-to-needle and onset-to-needle time did not differ between timeframes as well as between network models. However, data were available for few studies, and the door-to-recanalization time was only available for mothership-based studies. Therefore, how a delay in onset-to-door can translate into longer onset-to-treatment time during the pandemic has yet to be defined. To this regard, it seems critical to organize both COVID-free and COVID-positive pathways for stroke care to deliver treatment with the same time metrics regardless of the infective status of the patient.^4,8,11^ Brain imaging represents a critical stage to limit delays, and availability of advanced CT imaging in both pathways might be crucial to limit door-to-scan time.^4,50^

Limitations of this meta-analysis can be seen in the heterogeneity of the study included, which span geographically and in time, following the first pandemic wave. However, the variation in stroke admissions and treatments is in line with reports from national-level surveys,^44,51^ lending weight to our results. The local changes to the availability of stroke beds might have impacted on stroke care, but our approach, considering stroke admissions and relative rates of reperfusion treatments, limited the bias in our results. A second limitation derives from the stroke network model adopted, which was not available in some studies. To this extent, sensitivity analysis has been provided to support the findings, with little differences emerging for main estimates. Third, onset-to-needle time was available for few studies, preventing the identification of subtle delays in treatment delivery. However, results seem to have little heterogeneity, and the adherence to the rate of reperfusion treatments provided during normal conditions also during COVID-period suggests that little if no impact remained from pre-hospital delay on treatment delivery. Fourth limitation, our systematic review did not include treatment outcome, a limitation that limits our conclusions to stroke networks rather than on stroke care. However, preliminary findings suggests that, when treatment delivery matches standards, the benefit on functional outcome is maintained.^9,52^ Finally, our results derived from the first pandemic wave only, therefore representing a “worse-case scenario” which might not be applicable to future waves. Overall, as we are still facing and are expected to face in the near future other pandemic waves, with further personal and medical burden, the picture emerging from this meta-analysis lends support to our efforts to ensure high-quality acute stroke care in global healthcare systems.

## Conclusions

The main finding of this meta-analysis is that, despite contraction in admissions, stroke networks have been able to deliver similar rates of treatment to those provided in pre-pandemic period, with similar timing, with both D&S and mothership models. Such findings suggest that stroke networks can deal with the pandemic wave, and that neurologists should advocate with local institutions to allocate appropriate resources to keep stroke networks in function, or adapt to local circumstances. Few studies included in this systematic review reported that Healthcare Authorities refrained from limiting the resources and capacity of stroke units.^4,27,30^ The results of this meta-analysis are critical to sustain and encourage such attitude.

## Supplemental Material

sj-pdf-1-wso-10.1177_17474930211041202 - Supplemental material for Stroke network performance during the first COVID-19 pandemic stage: A meta-analysis based on stroke network models Click here for additional data file. Supplemental material, sj-pdf-1-wso-10.1177_17474930211041202 for Stroke network performance during the first COVID-19 pandemic stage: A meta-analysis based on stroke network models by Michele Romoli, Paolo Eusebi, Stefano Forlivesi, Mauro Gentile, Fabrizio Giammello, Laura Piccolo, David Giannandrea, Simone Vidale, Marco Longoni, Matteo Paolucci, Jessica Hsiao, Emily Sayles, Leonard LL Yeo, Espen Saxhaug Kristoffersen, Angel Chamorro, Liqun Jiao, Pooja Khatri, Georgios Tsivgoulis, Maurizio Paciaroni and Andrea Zini in International Journal of Stroke
