# Supplementary material for: Stroke network performance during the first COVID-19 pandemic stage: A meta-analysis based on stroke network models
Source: Int J Stroke. 2021 Aug 28;16(7):771–83. doi: 10.1177/17474930211041202 (PMC8521356; doi:10.1177/17474930211041202)
Supplement: sj-pdf-1-wso-10.1177_17474930211041202 - Supplemental material for Stroke network performance during the first COVID-19 pandemic stage: A meta-analysis based on stroke network models [file sj-pdf-1-wso-10.1177_17474930211041202.pdf]

SUPPLEMENTAL MATERIAL

Literature search string

Supplemental material – Figure I. PRISMA flow-chart.

Supplemental material – Table I. Excluded studies.

Supplemental material – Table II. Bias assessment.

Supplemental material – Table III. Meta-regression analysis for time of rescue and reperfusion depending on stroke network model.

Supplemental material – Figure II. Funnel plots for studies reporting incidence rate ratio (IRR) of stroke admissions (top), intravenous thrombolysis rate ratio (center) and endovascular treatment rate ratio (bottom) in the two timeframes.

Supplemental material – Figure III. Combinatorial meta-analysis showing homogeneous distribution and no cluster segregation for weekly stroke admissions incidence rate ratio (IRR, top), weekly intravenous thrombolysis rate ratio (center), and endovascular thrombectomy (bottom).

Supplemental material – Figure IV. Meta-analysis of intravenous thrombolysis incidence (IVT-IRR, top) and endovascular thrombectomy incidence rate ratio (EVT-IRR, bottom), comparing stroke admissions during COVID-period vs control-period, depending on stroke network model.

Supplemental material – Figure V. Variations in National Institutes of Health Stroke Scale (NIHSS) score between COVID-period and control-period, depending on stroke network model (top). Sensitivity analysis excluding studies with unknown stroke network model (centre), and restricting analysis to studies with prospective design only (bottom).

Supplemental material – Figure VI. Variations in the proportion of people with large vessel occlusion among those presenting with acute ischemic stroke during the timeframes considered (COVID-period vs control-period), depending on stroke network model.

Supplemental material – Figure VII. Sensitivity analysis for proportion of patients undergoing intravenous thrombolysis (IVT) among those admitted with acute ischemic stroke during the timeframes considered (COVID-period vs control-period), removing studies with unknown stroke network model (top), or including only prospective studies (bottom).

Supplemental material – Figure VIII. Sensitivity analysis for proportion of patients undergoing endovascular treatment (EVT) among those admitted with acute ischemic stroke during the timeframes considered (COVID-period vs control-period), removing studies with unknown stroke network model (top), or including only prospective studies (bottom).

Supplemental material – Figure IX. Sensitivity analysis for onset-to-door time across the timeframes considered, removing studies with unknown stroke network model (top), or including only prospective studies (bottom).

Supplemental material – Figure X. Variation in door-to-scan time depending on timeframes considered (COVID-19 period vs control period), with studies grouped for stroke network model used.

Supplemental material – Figure XI. Variation in door-to-needle time in patients receiving intravenous thrombolysis (IVT) for acute ischemic stroke during COVID-19 and control period, depending on stroke network model adopted.

Supplemental material – Figure XII. Variations in onset-to-needle time across the timeframes considered, depending on stroke network model organization.

Supplemental material – Figure XIII. Variations in door-to-groin time across the timeframes considered depending on stroke network model organization.

Supplemental material – Figure XIV. Sensitivity analysis for variations in door-to-groin time across the timeframes considered, removing studies with unknown stroke network model (top), or including only prospective studies (bottom).

Supplemental material – Figure XV. Variations in door-to-recanalization time across the timeframes considered depending on stroke network model organization.

Literature search string

(“cerebrovascular disease” [All Fields] OR “stroke” [All FIelds] OR cerebrovascular accident [All Fields]) AND ("coronavirus"[MeSH Terms] OR "coronavirus"[All Fields] OR "COVID-19"[Supplemental Concept] OR "severe acute respiratory syndrome coronavirus 2"[Supplemental Concept] OR COVID[All Fields] OR "covid-19"[All Fields] OR "covid19"[All Fields] OR "novel coronavirus"[All Fields] OR nCoV[All Fields] OR "SARS-CoV2"[All Fields]); until 8/10/2020, only 2019 or 2020 published papers, English language only.

Supplemental material – Figure I. PRISMA flow-chart.

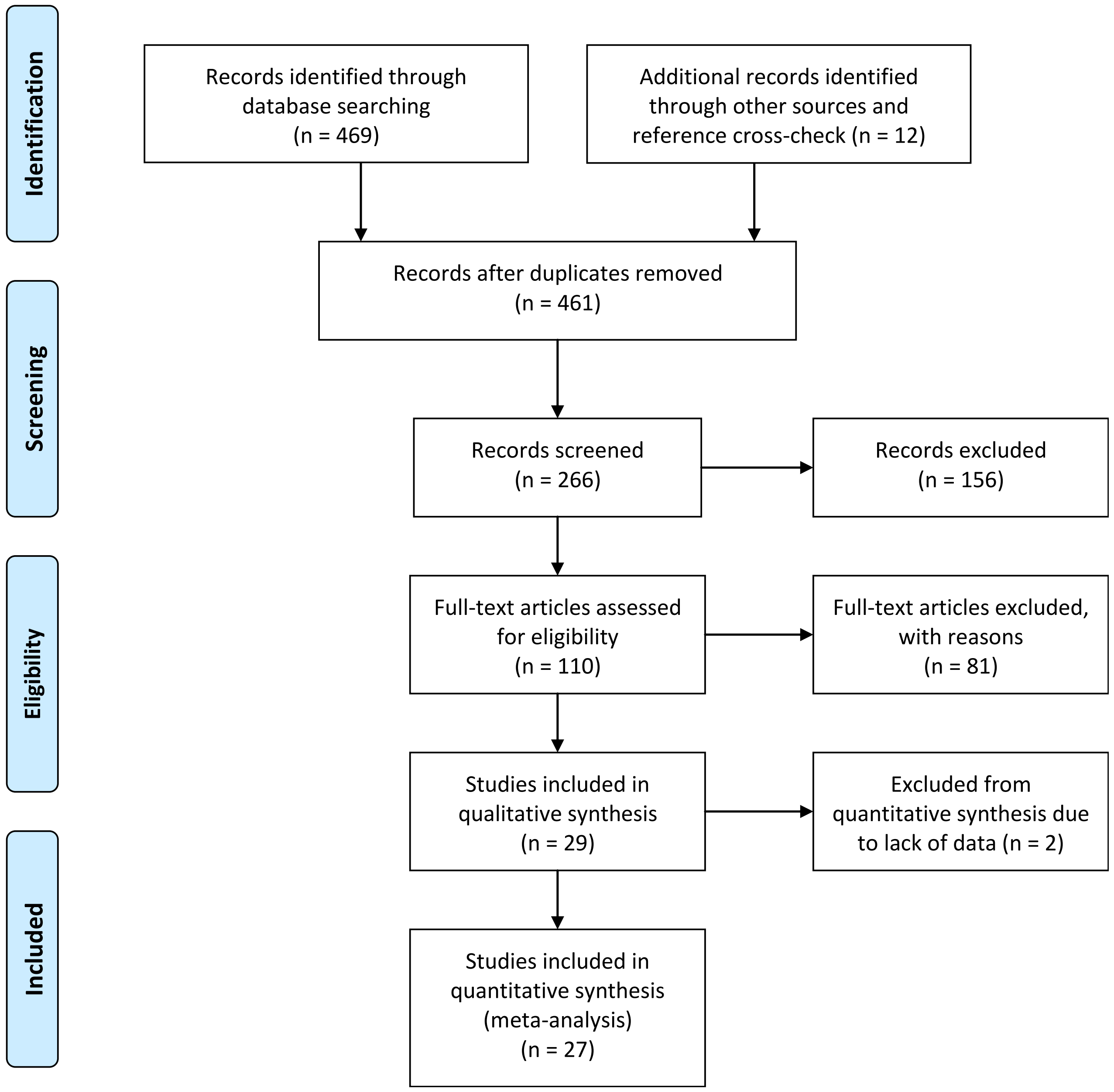

Supplemental material – Table I. Excluded studies

Author	Year	Reason for exclusion
Aggarwal[1]	2020	No data, association of stroke with/without COVID
Al Kasab[2]	2020	Letter to the editor, no data
Al-Ani[3]	2020	Review on thrombosis risk in COVID
Alqahtani[4]	2020	Description of code stroke protocol, no data
Azarpazhooh[5]	2020	No data on stroke admissions/incidence
Beghi[6]	2020	Review only, no data
Belani[7]	2020	No data, only COVID cohort
Bersano[8]	2020	Recommendation only, no data
Bhaskar[9]	2020	Guidelines, no data
Bress Bullrich[10]	2020	Unclear identification of stroke (ischemic/hemorrhagic)
Cantador[11]	2020	No data, only COVID cohort
Cavallieri[12]	2020	Case report
Chaturvedi[13]	2020	Recommendation only, no data
Cummings[14]	2020	Unclear identification of stroke
Dafer[15]	2020	No data on stroke incidence/treatment
D'Anna[16]	2020	No data, only COVID cohort
de Sousa[17]	2020	Editorial, no data
Derraz[18]	2020	Editorial, no data
Escalard[19]	2020	Case series, EVT in COVID+ patients
Frisullo[20]	2020	No data, letter
Frisullo[21]	2020	No data
Fuentes[22]	2020	Survey, no data
Gao[23]	2020	No data on stroke incidence/treatment
Goldberg[24]	2020	Case report
Goyal[25]	2020	No data
Gulko[26]	2020	Case report
Hajdu[27]	2020	Multiple timepoints, no raw data, EVT only
Hecht[28]	2020	No data on stroke incidence/treatment
Holt[29]	2020	Not pertinent (addressed new-onset atrial fibrillation)
Hoyer[30]	2020	No data (TIA/stroke admissions considered; data not provided)
Immovilli[31]	2020	No data, only COVID cohort
Jillela[32]	2020	No control group, case series
Jin[33]	2020	MERS, not SARS-CoV2
Kansagra[34]	2020	No data on stroke incidence/treatment
Katz[35]	2020	No data, only COVID cohort
Khosravani[36]	2020	Description of protected code stroke, no data
Lange[37]	2020	Unclear identification of stroke (ischemic/hemorrhagic)
Leira[38]	2020	Review, no original data
Leslie-Mazwi[39]	2020	Review, no original data
Li[40]	2020	No data on stroke incidence/treatment
Lima[41]	2020	Case report
Lumley-Holmes[42]	2020	Letter, no data
Majidi[43]	2020	No temporal trend comparison
Maldonad Slootjes[44]	2020	Case report
Mansoor[45]	2020	case report
Markus[46]	2020	Editorial, no data
Masroor[47]	2020	Case report
Merkler[48]	2020	No data, only COVID cohort
Meyer[49]	2020	No data
Mishra[50]	2020	Letter to the editor, no data
Mohamud[51]	2020	Case series on COVID, no data
Morelli[52]	2020	Control period not defined
Morelli[53]	2020	Reply, no data
Nguyen[54]	2020	Recommendation only, no data
Ntaios[55]	2020	No temporal trend comparison
Ospel[56]	2020	Recommendation only, no data
Pasarikovski[57]	2020	No raw data
Paterson[58]	2020	No raw data
Pedicelli[59]	2020	No raw data
Qureshi [60]	2020	Recommendation only
Qureshi [61]	2020	Not pertinent
Qureshi[62]	2020	No data on stroke incidence/treatment

Reddy[63]	2020	Review only, no data
Rodriguez-Pardo [64]	2020	Recommendation only
Saggese[65]	2020	Case report
Salahuddin[66]	2020	Recommendation only
Sangalli[67]	2020	Case series
Shahjouei[68]	2020	No control group, hospitalized patients only
Sheth[69]	2020	No data
Sierra-Hidalgo[70]	2020	No data, only COVID cohort
Sweid[71]	2020	Case series, COVID cohort
Sylaja[72]	2020	Survey, no data
Tsivgoulis[73]	2020	Editorial
Valdes Valderrama[74]	2020	Case report
Varatharaj[75]	2020	Survey of acute neurological and psychiatric complications of COVID-19
Venketasubramanian[76]	2020	Editorial, no data
Wang[77]	2020	Commentary, no data
Yaeger[78]	2020	No data, only COVID cohort
Yaghi[79]	2020	Stroke patients with COVID vs without COVID vs historical cohort
Zhai[80]	2020	Case report
Zhao[81]	2020	Editorial, no data

Supplemental material – Table II. Bias assessment.

Author	Year	1) Representativeness of the exposed cohort	2) Selection of the non exposed cohort	3) Ascertainment of exposure	4) Demonstration that outcome of interest was not present at start of study	5) comparability of the cohorts included	6) Assessment of outcome	7) Length of follow-up	8) Adequacy of follow up of cohorts	**Global score**	**Risk of bias**
Agarwal[82]	2020	1	1	1	1	1	1	1	1	8	low
Baracchini[83]	2020	0	0	1	1	0	0	0	0	2	high
De Havenon[84]	2020	1	1	1	1	1	1	1	1	8	low
Diegoli[85]	2020	1	1	1	1	1	1	1	1	8	low
Esenwa[86]	2020	1	1	0	1	1	1	1	1	7	low
Hsiao[87]	2020	1	1	1	1	1	1	1	1	8	low
Huang[88]	2020	1	1	1	1	2	1	1	1	9	low
Jasne[89]	2020	1	1	1	1	1	1	1	1	8	low
Kerleroux[90]*	2020	1	1	1	1	1	1	0	1	7	low
Mehrpour[91]	2020	0	1	1	1	1	1	1	1	7	low
Montaner[92]	2020	1	1	1	1	0	1	0	0	5	moderate
Naccarato[93]	2020	1	1	1	1	1	1	1	1	8	low
Nguyen-Huynh[94]	2020	1	1	1	1	1	1	1	1	8	low
Onteddu[95]	2020	1	1	0	1	1	1	1	1	7	low
Paliwal[96]	2020	1	1	1	1	2	1	1	1	9	low
Pandey[97]	2020	1	1	1	1	1	1	1	1	8	low
Perry[98]	2020	1	1	0	0	2	0	0	0	4	moderate
Pop[99]	2020	1	1	1	1	1	1	0	1	7	low
Rudilosso[100]	2020	1	1	0	1	1	1	1	1	7	low
Saxhaug Kristoffersen[101]	2020	1	1	1	1	2	1	1	1	9	low
Schirmer[102]	2020	1	1	1	1	0	1	1	1	7	low
Siegler[103]	2020	1	1	1	1	1	1	1	1	8	low
Strasser[104]	2020	1	1	1	1	1	1	1	1	8	low
Tejada Meza[105]	2020	1	1	1	1	1	1	1	1	8	low
Tejada Meza[106]	2020	1	1	1	1	1	1	0	1	7	low
Teo[107]	2020	1	1	1	1	1	0	1	1	7	low
Yang[108]	2020	1	1	1	1	1	1	0	1	7	low
Zhao[109]*	2020	1	1	0	1	1	0	0	0	4	moderate
Zini[110]	2020	1	1	1	1	1	1	1	1	8	low

Supplemental material – Table III. Meta-regression analysis for time of rescue and reperfusion depending on stroke network model.

Legend. CI=confidence interval; NA=not available.

Time and model	Estimate (95%CI)(minutes)	p-value
**Onset-to-door**		
Drip&ship	reference	//
Mothership	-44.3 (-85.3 - -3.2)	0.03
Unknown	-17.8 (-115.3 - +79.6)	0.7
**Door-to-groin**		
Drip&ship	reference	//
Mothership	-14.4 (-10.5 - +39.23)	0.26
Unknown	10.2 (-23.4 - +43.7)	0.55
**Onset-to-needle**		
Drip&ship	reference	//
Mothership	-36.0 (-142.9 - +70.4)	0.50
Unknown	NA	NA

Supplemental material – Figure II. Funnel plots for studies reporting incidence rate ratio (IRR) of stroke admissions (top), intravenous thrombolysis rate ratio (center) and endovascular treatment rate ratio (bottom) in the two timeframes.

Legend. Light blue: $p<0.05$; Dark blue: $p<0.01$; Dots: studies.

Stroke admissions IRR

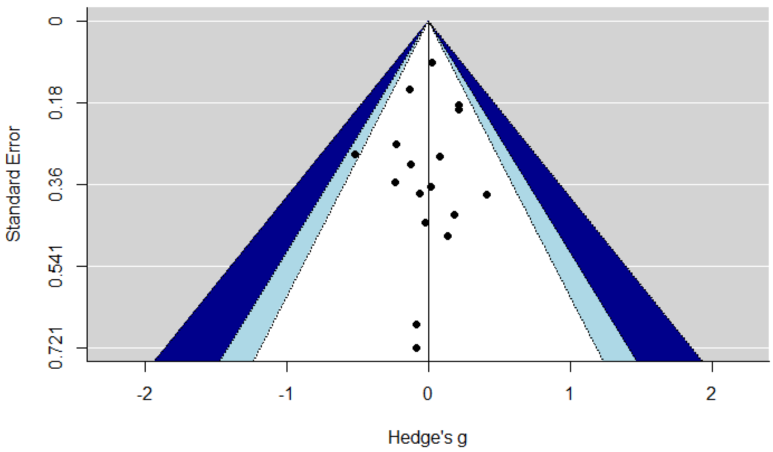

Intravenous thrombolysis IRR

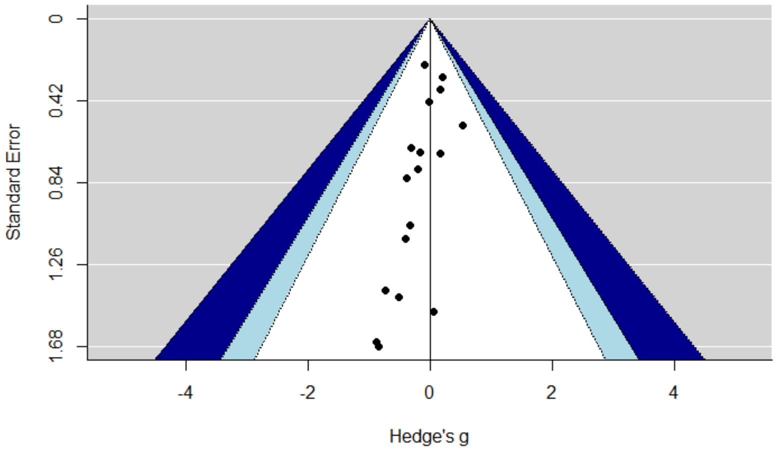

Endovascular thrombectomy IRR

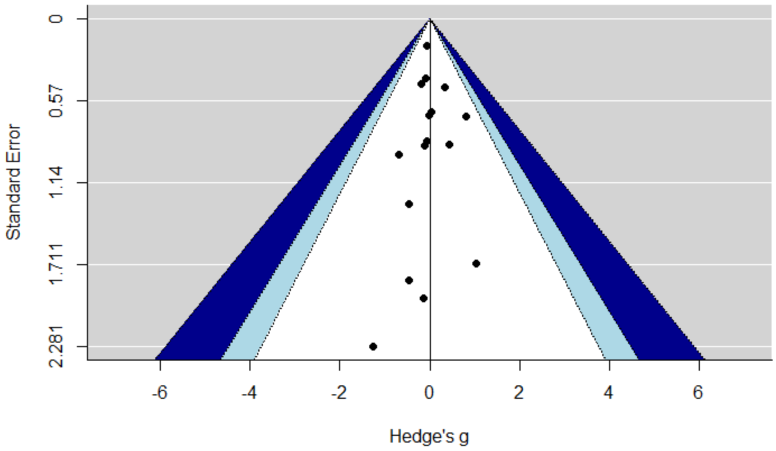

Supplemental material – Figure III. Combinatorial meta-analysis showing homogeneous distribution and no cluster segregation for weekly stroke admissions incidence rate ratio (IRR, top), weekly intravenous thrombolysis rate ratio (center), and endovascular thrombectomy (bottom).

Stroke admissions IRR

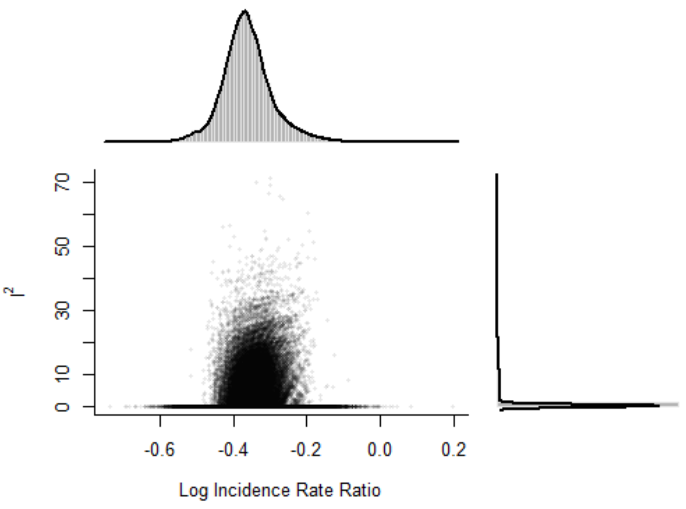

Intravenous thrombolysis-IRR

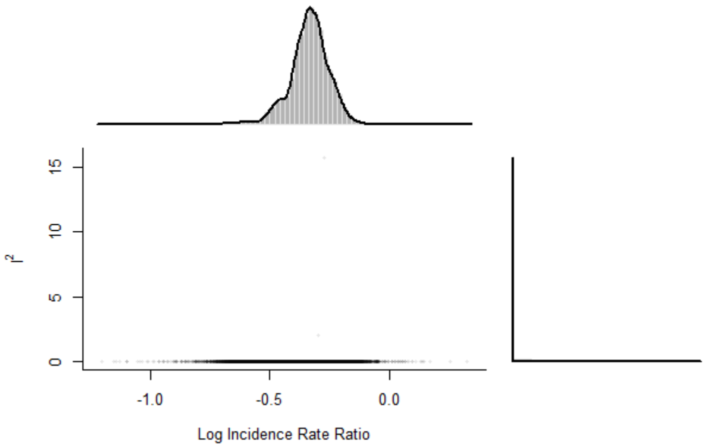

Endovascular thrombectomy-IRR

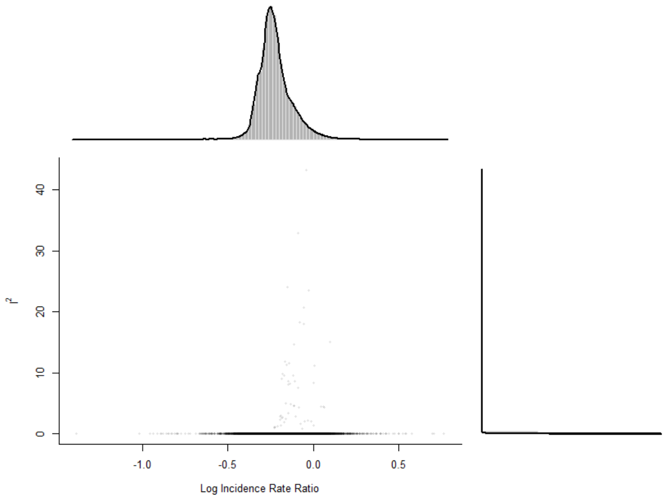

Supplemental material – Figure IV. Meta-analysis of intravenous thrombolysis incidence (IVT-IRR, top) and endovascular thrombectomy incidence rate ratio (EVT-IRR, bottom), comparing stroke admissions during COVID-period vs control-period, depending on stroke network model.

Legend. CI: confidence interval; EVT: endovascular thrombectomy; IRR: incidence rate ratio (equals treatment rate during COVID-19 period/treatment rate during control period); IVT: intravenous thrombolysis; RE: random effect.

IVT-IRR

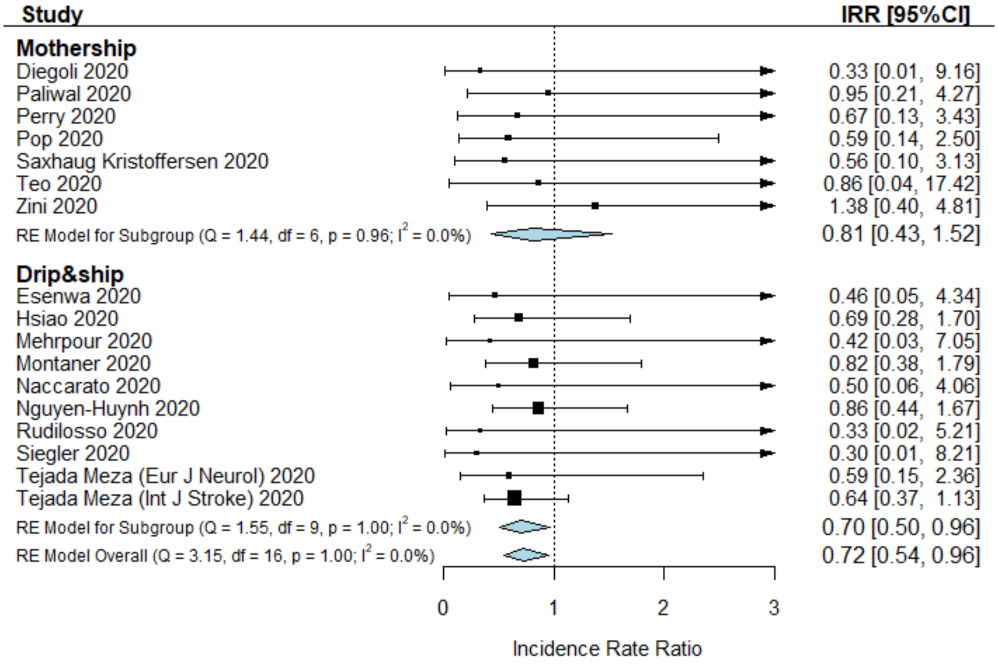

EVT-IRR

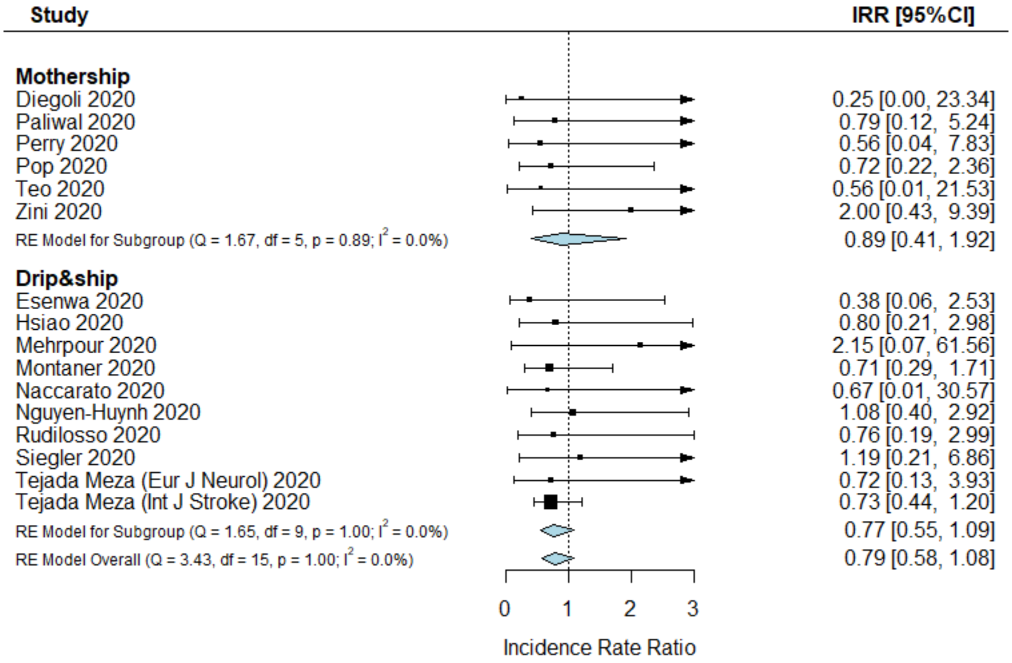

Supplemental material – Figure V. Variations in National Institutes of Health Stroke Scale (NIHSS) score between COVID-period and control-period, depending on stroke network model (top). Sensitivity analysis excluding studies with unknown stroke network model (centre), and restricting analysis to studies with prospective design only (bottom).

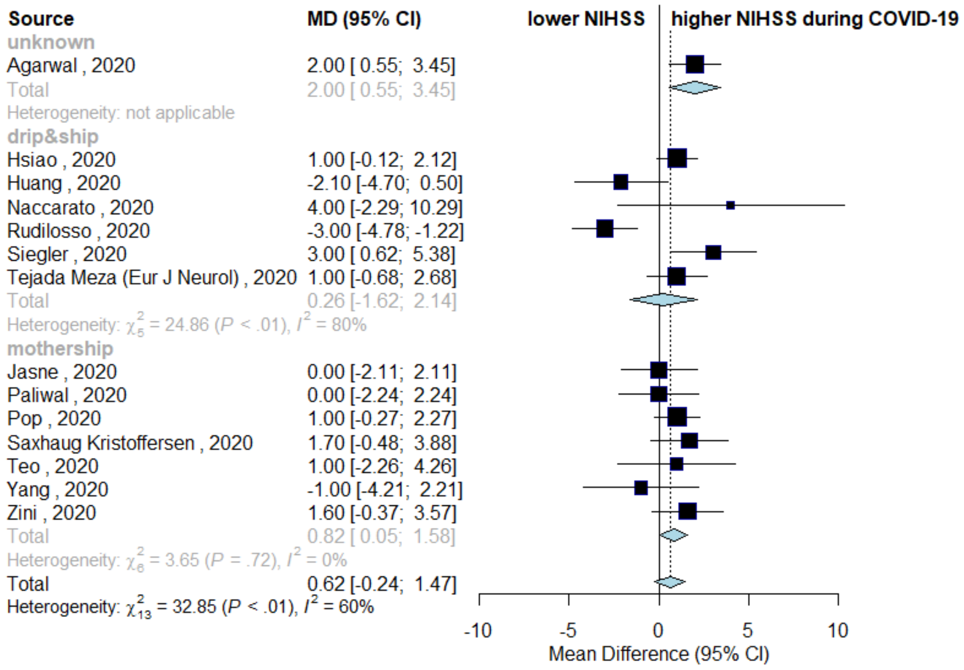

Sensitivity analysis excluding studies with unknown stroke network model

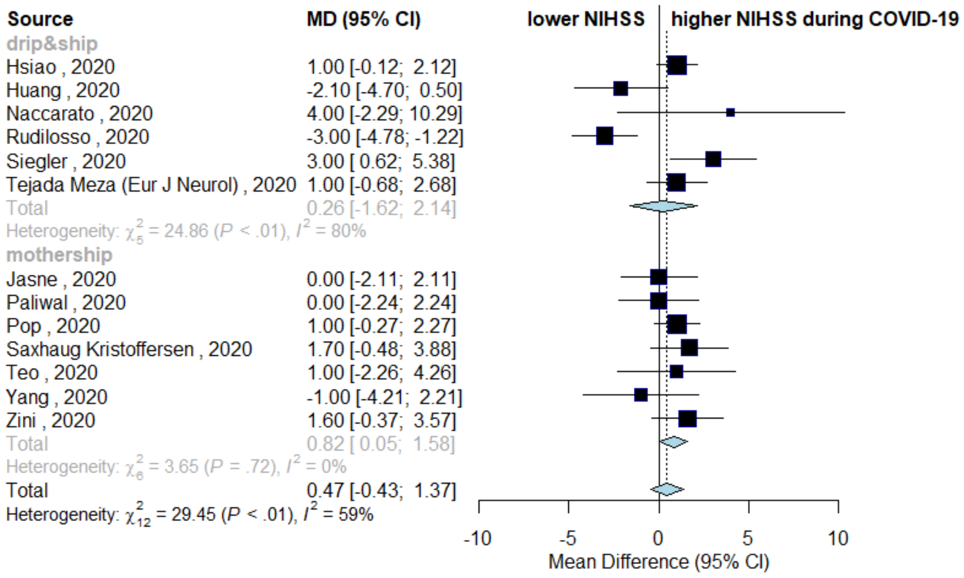

Sensitivity analysis with prospective design-only studies

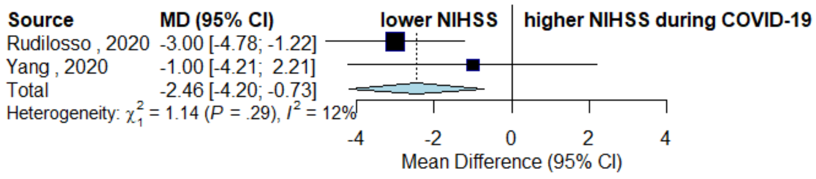

Supplemental material – Figure VI. Variations in the proportion of people with large vessel occlusion among those presenting with acute ischemic stroke during the timeframes considered (COVID-period vs control-period), depending on stroke network model.

Legend. CI=confidence interval; COVID=period of SARS-CoV-2 pandemic (coronavirus pandemic); RR=relative risk.

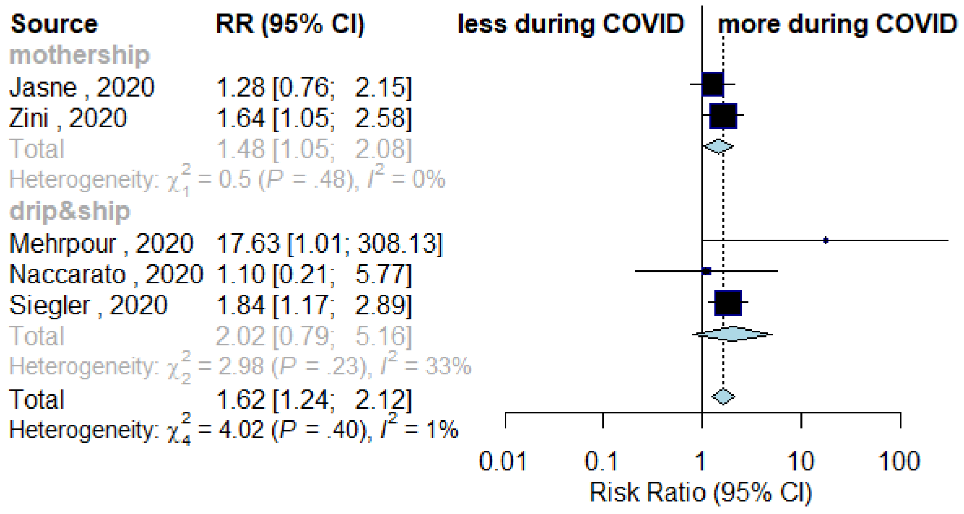

Supplemental material – Figure VII. Sensitivity analysis for proportion of patients undergoing intravenous thrombolysis (IVT) among those admitted with acute ischemic stroke during the timeframes considered (COVID-period vs control-period), removing studies with unknown stroke network model (top), or including only prospective studies (bottom).

Legend. CI=confidence interval; COVID=period of SARS-CoV-2 pandemic (coronavirus pandemic); RR=relative risk.

Sensitivity analysis excluding studies with unknown stroke network model

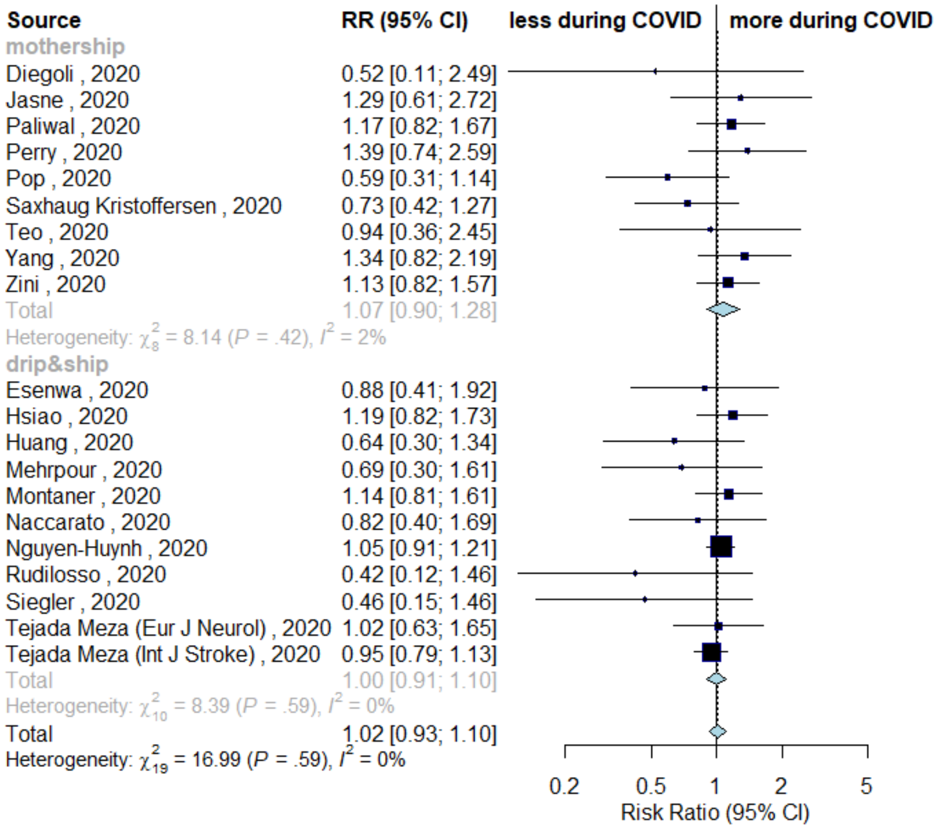

Sensitivity analysis with prospective studies-only

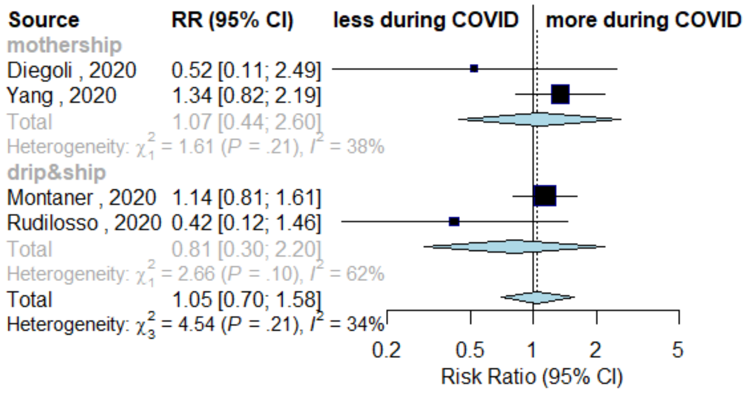

Supplemental material – Figure VIII. Sensitivity analysis for proportion of patients undergoing endovascular treatment (EVT) among those admitted with acute ischemic stroke during the timeframes considered (COVID-period vs control-period), removing studies with unknown stroke network model (top), or including only prospective studies (bottom).

Legend. CI=confidence interval; COVID=period of SARS-CoV-2 pandemic (coronavirus pandemic); RR=relative risk.

Sensitivity analysis excluding studies with unknown stroke network model

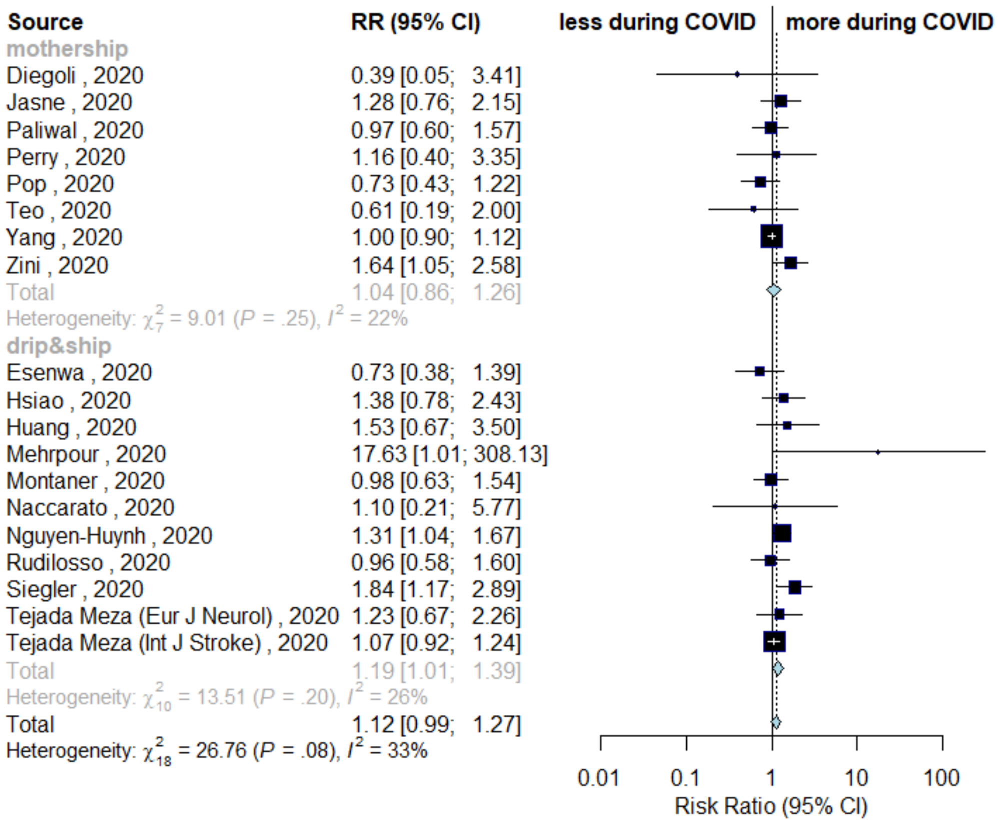

Sensitivity analysis with prospective studies-only

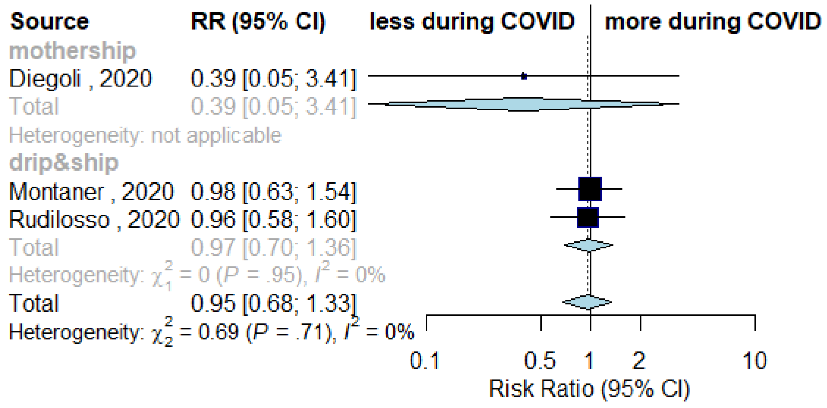

Supplemental material – Figure IX. Sensitivity analysis for onset-to-door time across the timeframes considered, removing studies with unknown stroke network model (top), or including only prospective studies (bottom).

Legend. CI=confidence interval; COVID-19=period of SARS-CoV-2 pandemic (coronavirus pandemic); MD=mean difference.

Sensitivity analysis excluding studies with unknown stroke network model

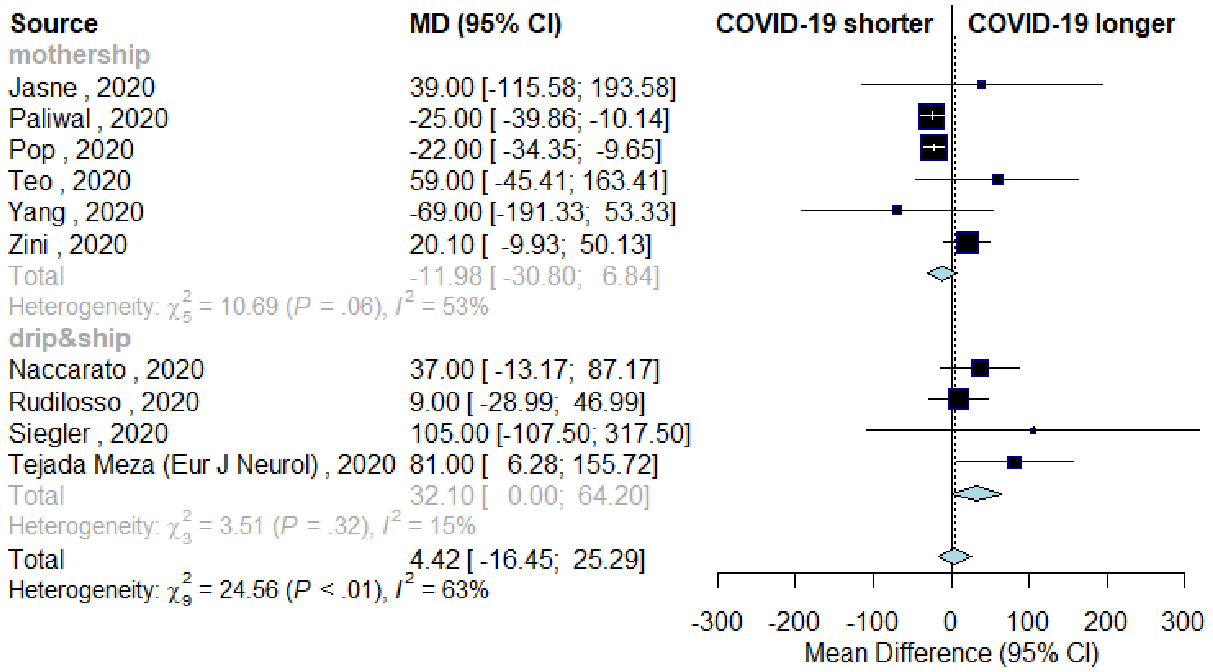

Sensitivity analysis with prospective studies-only

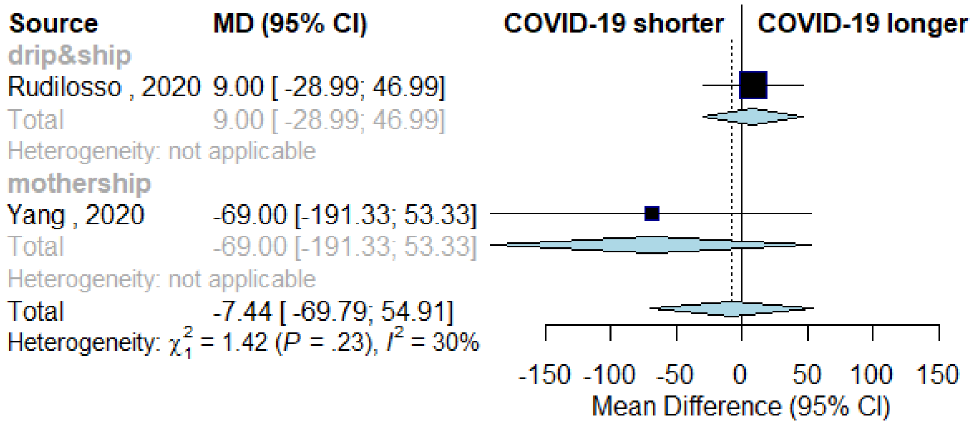

Supplemental material – Figure X. Variation in door-to-scan time depending on timeframes considered (COVID-19 period vs control period), with studies grouped for stroke network model used.

Legend. CI=confidence interval; COVID-19=period of SARS-CoV-2 pandemic (coronavirus pandemic); MD=mean difference.

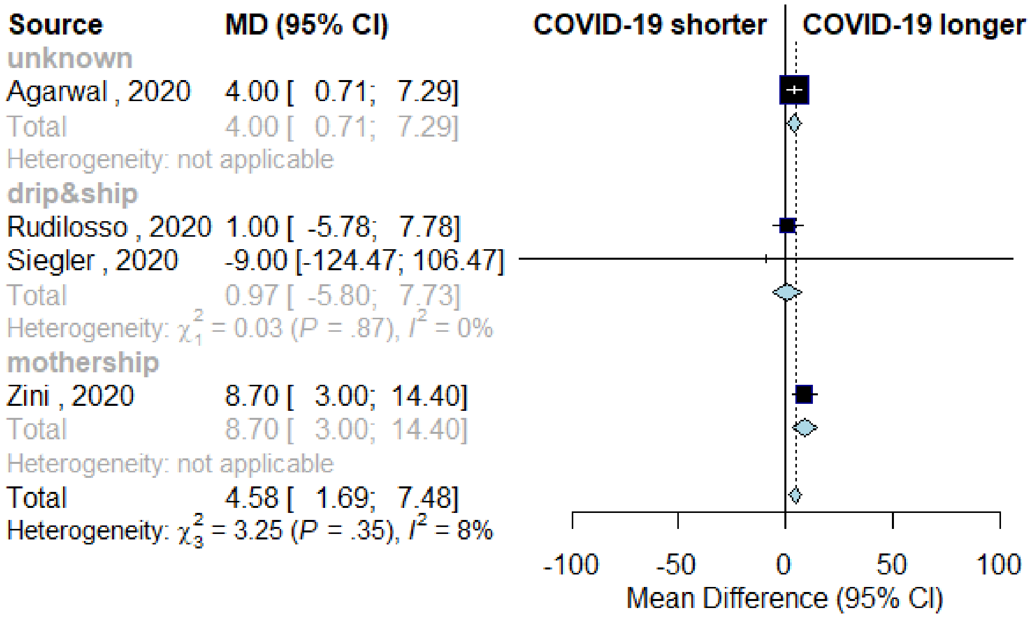

Supplemental material – Figure XI. Variation in door-to-needle time in patients receiving intravenous thrombolysis (IVT) for acute ischemic stroke during COVID-19 and control period, depending on stroke network model adopted.

Legend. CI=confidence interval; COVID-19=period of SARS-CoV-2 pandemic (coronavirus pandemic); MD=mean difference.

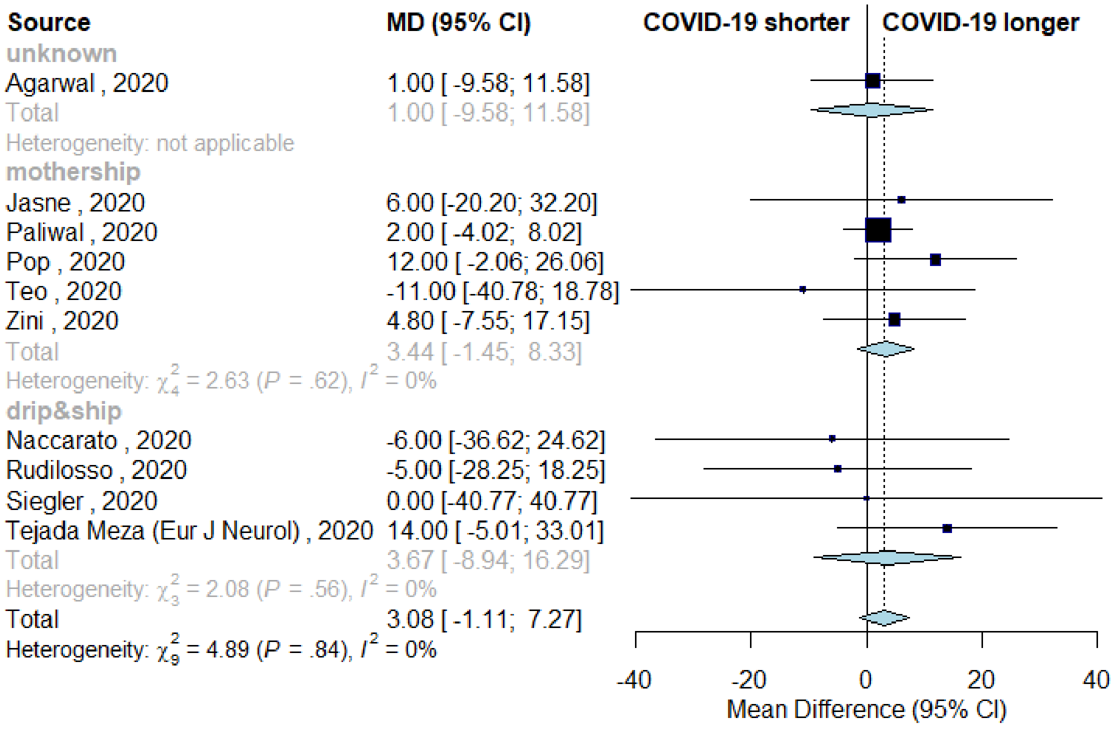

Supplemental material – Figure XII. Variations in onset-to-needle time across the timeframes considered, depending on stroke network model organization.

Legend. CI=confidence interval; COVID-19=period of SARS-CoV-2 pandemic (coronavirus pandemic); MD=mean difference.

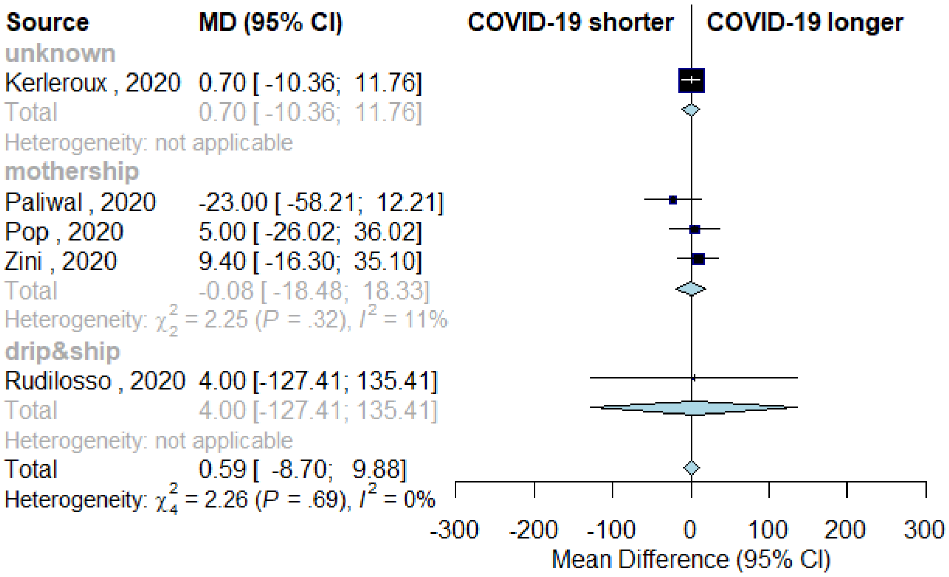

Supplemental material – Figure XIII. Variations in door-to-groin time across the timeframes considered depending on stroke network model organization.

Legend. CI=confidence interval; COVID-19=period of SARS-CoV-2 pandemic (coronavirus pandemic); MD=mean difference.

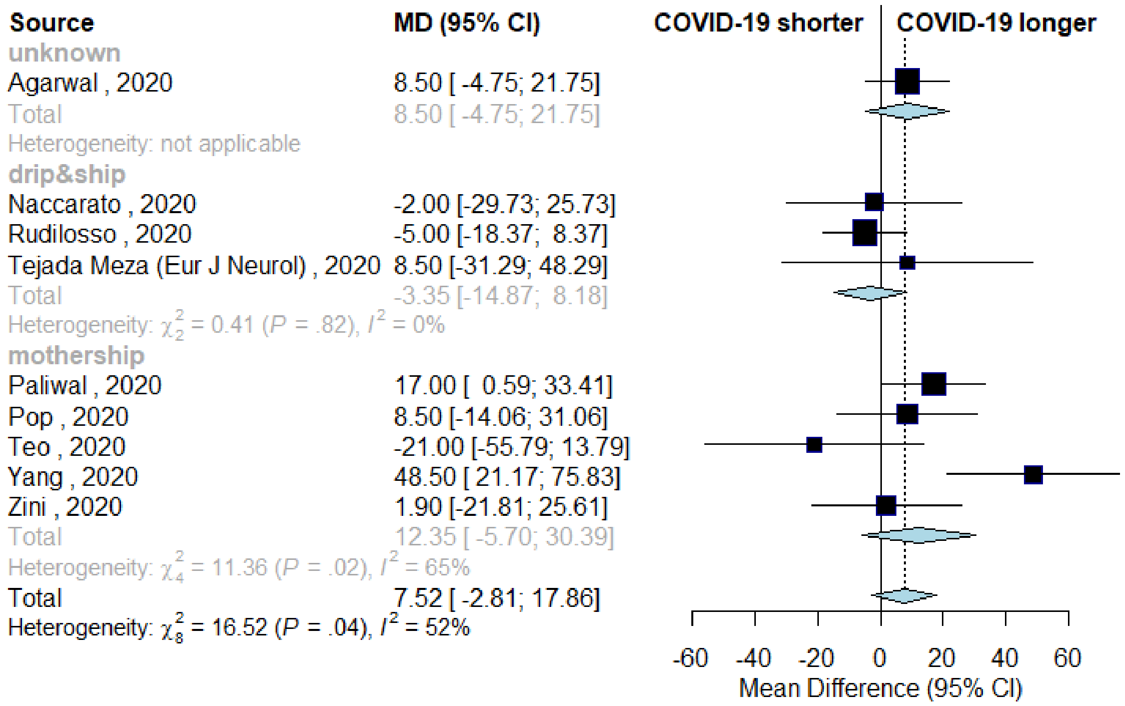

Supplemental material – Figure XIV. Sensitivity analysis for variations in door-to-groin time across the timeframes considered, removing studies with unknown stroke network model (top), or including only prospective studies (bottom).

Legend. CI=confidence interval; COVID-19=period of SARS-CoV-2 pandemic (coronavirus pandemic); MD=mean difference

Sensitivity analysis excluding studies with unknown network organization

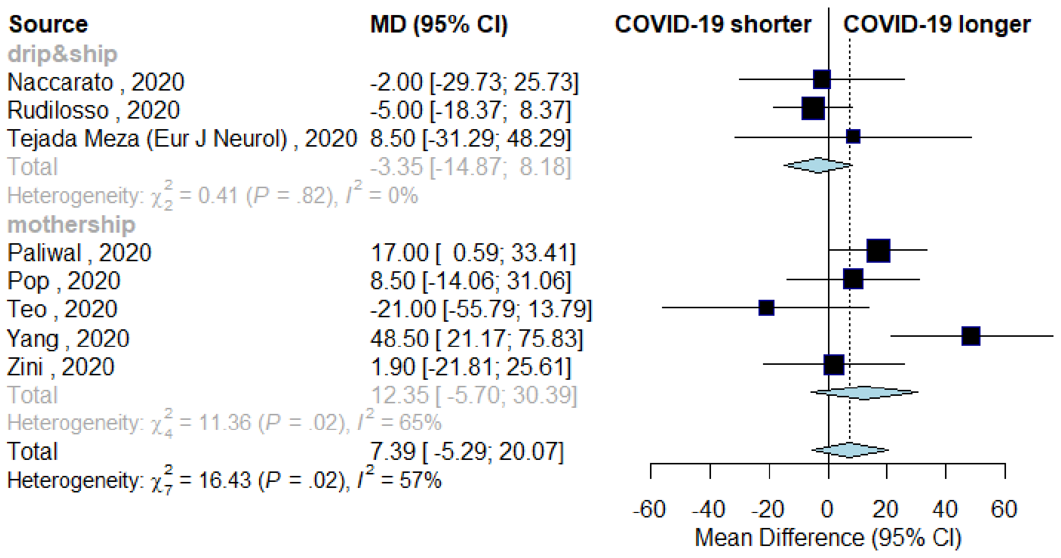

Sensitivity analysis with prospective studies-only

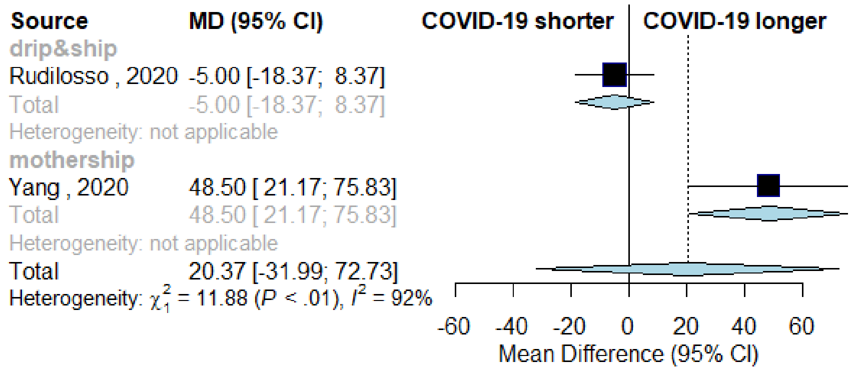

Supplemental material – Figure XV. Variations in door-to-recanalization time across the timeframes considered depending on stroke network model organization.

Legend. CI=confidence interval; COVID-19=period of SARS-CoV-2 pandemic (coronavirus pandemic); MD=mean difference.

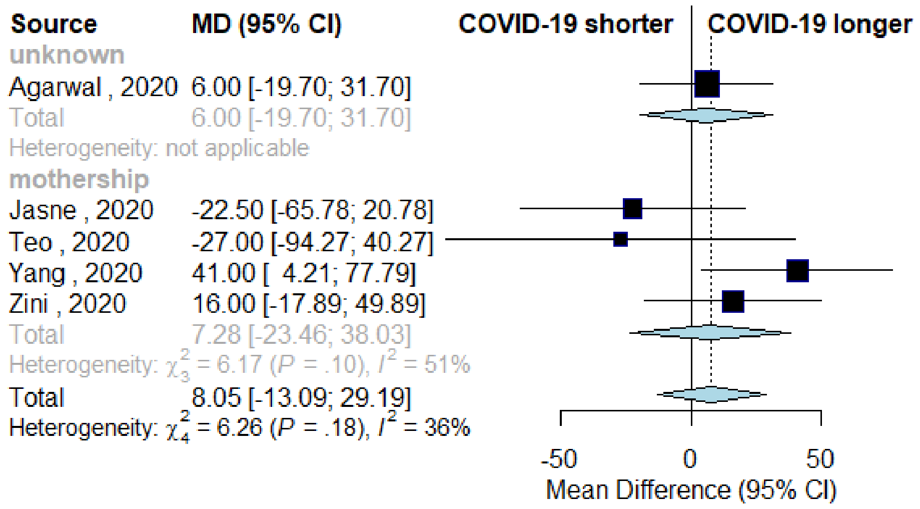

References

1 Aggarwal G, Lippi G, Michael Henry B. Cerebrovascular disease is associated with an increased disease severity in patients with Coronavirus Disease 2019 (COVID-19): A pooled analysis of published literature. *Int J Stroke* 2020;**0**:1–5. doi:10.1177/1747493020921664

2 Al Kasab S, Almallouhi E, Spiotta AM. Acute Stroke Management During the Coronavirus Disease 2019 (COVID-19) Pandemic: From Trough of Disillusionment to Slope of Enlightenment. *World Neurosurg* 2020;**2019**:2019–20. doi:10.1016/j.wneu.2020.04.153

3 Al-Ani F, Chehade S, Lazo-Langner A. Thrombosis risk associated with COVID-19 infection. A scoping review. *Thromb Res* 2020;**192**:152–60. doi:10.1016/j.thromres.2020.05.039

4 Alqahtani SA, Alnaami I, Alhazzani A. Protected Stroke Mechanical Thrombectomy Code During the Coronavirus (COVID-19) Pandemic: Southwestern Part of Saudi Arabia Stroke Unit Local Protocol. *Cureus* 2020;**12**:4–9. doi:10.7759/cureus.7808

5 Azarpazhooh MR, Morovatdar N, Avan A, *et al.* COVID-19 Pandemic and Burden of Non-Communicable Diseases: An Ecological Study on Data of 185 Countries. *J Stroke Cerebrovasc Dis* 2020;**29**:105089. doi:10.1016/j.jstrokecerebrovasdis.2020.105089

6 Beghi E, Feigin V, Caso V, *et al.* COVID-19 Infection and Neurological Complications: Present Findings and Future Predictions. *Neuroepidemiology* 2020;:1–6. doi:10.1159/000508991

7 Belani P, Schefflein J, Kihira S, *et al.* COVID-19 Is an Independent Risk Factor for Acute Ischemic Stroke. *Am J Neuroradiol* 2020;:1–4. doi:10.3174/ajnr.a6650

8 Bersano A, Kraemer M, Touzé E, *et al.* Stroke care during the Covid-19 pandemic: Experience from three large European countries. *Eur J Neurol* Published Online First: 2020. doi:10.1111/ene.14375

9 Bhaskar S, Sharma D, Walker AH, *et al.* Acute Neurological Care in the COVID-19 Era: The Pandemic Health System REsilience PROGRAM (REPROGRAM) Consortium Pathway. *Front Neurol* 2020;**11**:1–8. doi:10.3389/fneur.2020.00579

10 Bres Bullrich M, Fridman S, Mandzia JL, *et al.* COVID-19: Stroke Admissions, Emergency Department Visits, and Prevention Clinic Referrals. *Can J Neurol Sci* 2020;**1**:1–10. doi:10.1017/cjn.2020.101

11 Cantador E, Núñez A, Sobrino P, *et al.* Incidence and consequences of systemic arterial thrombotic events in COVID-19 patients. *J Thromb Thrombolysis* 2020;:1–5. doi:10.1007/s11239-020-02176-7

12 Cavallieri F, Marti A, Fasano A, *et al.* Prothrombotic state induced by COVID-19 infection as trigger for stroke in young patients: A dangerous association. *eNeurologicalSci* 2020;**20**:18–20. doi:10.1016/j.ensci.2020.100247

13 Chaturvedi S, Gorelick P. The interplay between COVID 19 and non-communicable diseases. *J Stroke Cerebrovasc Dis* 2020;:105104. doi:10.1016/j.jstrokecerebrovasdis.2020.105104

14 Cummings C, Almallouhi E, Al Kasab S, *et al.* Blacks Are Less Likely to Present With Strokes During the COVID-19 Pandemic. *Stroke* 2020;:3107–11. doi:10.1161/strokeaha.120.031121

15 Dafer RM, Osteraas ND, Biller J. Acute Stroke Care in the Coronavirus Disease 2019 Pandemic. *J Stroke Cerebrovasc Dis* 2020;:104881. doi:10.1016/j.jstrokecerebrovasdis.2020.104881

16 D’Anna L, Kwan J, Brown Z, *et al.* Characteristics and clinical course of Covid-19 patients admitted with acute stroke. *J Neurol* Published Online First: 2020. doi:10.1007/s00415-020-10012-4

17 Aguiar de Sousa D, Worp HB. Stroke care in Europe during the COVID-19 pandemic. *Eur J Neurol* 2020;**27**:1793–1793. doi:10.1111/ene.14401

18 Derraz I. Stroke Health Care Use and COVID-19. *AJNR Am J Neuroradiol* 2020;:6563. doi:10.3174/ajnr.A6563

19 Escalard S, Maïer B, Redjem H, *et al.* Treatment of Acute Ischemic Stroke due to Large Vessel

Occlusion With COVID-19. *Stroke* 2020;:1–4. doi:10.1161/strokeaha.120.030574

20 Frisullo G, De Belvis AG, Marca G Della, *et al.* Stroke integrated care pathway during COVID-19 pandemic. *Neurol Sci* 2020;:1–3. doi:10.1007/s10072-020-04480-9

21 Frisullo G, Brunetti V, Di Iorio R, *et al.* Effect of lockdown on the management of ischemic stroke: an Italian experience from a COVID hospital. *Neurol Sci* Published Online First: 2020. doi:10.1007/s10072-020-04545-9

22 Fuentes B, Alonso de Leciñana M, Calleja-Castaño P, *et al.* Impact of the COVID-19 pandemic on the organisation of stroke care. Madrid Stroke Care Plan. *Neurologia* Published Online First: 2020. doi:10.1016/j.nrl.2020.05.007

23 Gao Q. The epidemiological characteristics of an outbreak of 2019 novel coronavirus diseases (COVID-19) in China. *Zhonghua Liu Xing Bing Xue Za Zhi* 2020;**41**:145–51. doi:10.3760/cma.j.issn.0254-6450.2020.02.003

24 Goldberg MF, Goldberg MF. Cerebrovascular Disease in COVID-19. 2020;:1–3.

25 Goyal M, Ospel JM, Southerland AM, *et al.* Prehospital Triage of Acute Stroke Patients During the COVID-19 Pandemic. *Stroke* 2020;:1–5. doi:10.1161/strokeaha.120.030340

26 Gulko E, Gomes W, Ali S, *et al.* Acute Common Carotid Artery Bifurcation Thrombus: An Emerging Pattern of Acute Strokes in Patients with COVID-19? *Am J Neuroradiol* 2020;:1–2. doi:10.3174/ajnr.A6657

27 Hajdu SD, Pittet V, Puccinelli F, *et al.* Acute Stroke Management during the COVID-19 Pandemic: Does Confinement Impact Eligibility for Endovascular Therapy? *Stroke* 2020;**51**:2593–6. doi:10.1161/STROKEAHA.120.030794

28 Hecht N, Wessels L, Werft F-O, *et al.* Need for ensuring care for neuro-emergencies-lessons learned from the COVID-19 pandemic. *Acta Neurochir (Wien)* Published Online First: 2020. doi:10.1007/s00701-020-04437-z

29 Holt A, Gislason GH, Schou M, *et al.* New-onset atrial fibrillation: incidence, characteristics, and related events following a national COVID-19 lockdown of 5.6 million people. *Eur Heart J* 2020;:1–8. doi:10.1093/eurheartj/ehaa494

30 Hoyer C, Ebert A, Huttner HB, *et al.* Acute Stroke in Times of the COVID-19 Pandemic: A Multicenter Study. *Stroke* 2020;:STROKEAHA120030395. doi:10.1161/STROKEAHA.120.030395

31 Immovilli P, Terracciano C, Zaino D, *et al.* Stroke in COVID-19 patients—A case series from Italy. *Int J Stroke* 2020;**0**:1–2. doi:10.1177/1747493020938294

32 Jillella D V., Janocko NJ, Nahab F, *et al.* Ischemic stroke in COVID-19: An urgent need for early identification and management. *PLoS One* 2020;**15**:e0239443. doi:10.1371/journal.pone.0239443

33 Jin C De, Kim MH, Lee KM, *et al.* Less Myocardial Infarction and Stroke Hospitalizations During Middle East Respiratory Syndrome Coronavirus Epidemic in Korea. *Am J Cardiol* 2020;**132**:176–8. doi:10.1016/j.amjcard.2020.06.065

34 Kansagra AP, Goyal MS, Hamilton S, *et al.* Collateral Effect of Covid-19 on Stroke Evaluation in the United States. *N Engl J Med* 2020;:9–10. doi:10.1056/nejmc2014816

35 Katz JM, Libman RB, Wang JJ, *et al.* Cerebrovascular Complications of COVID-19. *Stroke* 2020;**51**:e227–31. doi:10.1161/STROKEAHA.120.031265

36 Khosravani H, Rajendram P, Notario L, *et al.* Protected Code Stroke: Hyperacute Stroke Management During the Coronavirus Disease 2019 (COVID-19) Pandemic. *Stroke* 2020;**2019**:STROKEAHA120029838. doi:10.1161/STROKEAHA.120.029838

37 Lange SJ, Ritchey MD, Goodman AB, *et al.* Potential Indirect Effects of the COVID-19 Pandemic on Use of Emergency Departments for Acute Life-Threatening Conditions — United States, January–May 2020. *MMWR Morb Mortal Wkly Rep* 2020;**69**:795–800. doi:10.15585/mmwr.mm6925e2

38 Leira EC, Russman AN, Biller J, *et al.* Preserving stroke care during the COVID-19 pandemic: Potential issues and solutions. *Neurology* Published Online First: 2020. doi:10.1212/WNL.0000000000009713

39 Leslie-Mazwi TM, Fargen KM, Levitt M, *et al.* Preserving Access: A Review of Stroke Thrombectomy during the COVID-19 Pandemic. *AJNR Am J Neuroradiol* 2020;:1–6. doi:10.3174/ajnr.A6606

40 Li Y, Li M, Wang M, *et al.* Acute cerebrovascular disease following COVID-19: a single center, retrospective, observational study. *Stroke Vasc Neurol* 2020;:1–6. doi:10.1136/svn-2020-000431

41 LIMA CFC, HOLANDA JLB, PESSOA MSL, *et al.* Acute ischemic stroke in a patient with COVID-19. *Arq Neuropsiquiatr* 2020;**78**:454–5. doi:10.1590/0004-282x20200057

42 Lumley-Holmes J, Brake S, Docherty M, *et al.* Emergency ambulance services for heart attack and stroke during UK's COVID-19 lockdown. *Lancet* 2020;**395**:e93–4. doi:10.1016/S0140-6736(20)31031-X

43 Majidi S, Fifi JT, Ladner TR, *et al.* Emergent Large Vessel Occlusion Stroke During New York City's COVID-19 Outbreak. *Stroke* 2020;**51**:2656–63. doi:10.1161/STROKEAHA.120.030397

44 Maldonado Slootjes S, Hammer F, Leempoel J, *et al.* Acute stroke care during the COVID-19 pandemic: difficult, but not impossible! *Acta Neurol Belg* 2020;:2–3. doi:10.1007/s13760-020-01422-2

45 Mansour OY, Malik AM, Linfante I. Mechanical Thrombectomy of COVID-19 positive acute ischemic stroke patient: a case report and call for preparedness. *BMC Neurol* 2020;**20**:358. doi:10.1186/s12883-020-01930-x

46 Markus HS. EXPRESS: COVID-19 and Stroke - A Global World Stroke Organisation perspective. *Int J Stroke* Published Online First: 2020. doi:10.1177/1747493020923472

47 Masroor S. Collateral damage of COVID-19 pandemic: Delayed medical care. *J Card Surg* 2020;:2–4. doi:10.1111/jocs.14638

48 Merkler AE, Parikh NS, Mir S, *et al.* Risk of Ischemic Stroke in Patients with Covid-19 versus Patients with Influenza. *medRxiv Prepr Serv Heal Sci* 2020;**53**:1689–99. doi:10.1101/2020.05.18.20105494

49 Meyer D, Meyer BC, Rapp KS, *et al.* A Stroke Care Model at an Academic, Comprehensive Stroke Center During the 2020 COVID-19 Pandemic. *J Stroke Cerebrovasc Dis* 2020;:104927. doi:10.1016/j.jstrokecerebrovasdis.2020.104927

50 Mishra AK, George AA, Sargent J. Letter to the Editor Regarding "Acute Stroke Management During the Coronavirus Disease 2019 (COVID-19) Pandemic: From Trough of Disillusionment to Slope of Enlightenment". *World Neurosurg* 2020;**142**:551–2. doi:10.1016/j.wneu.2020.06.111

51 Mohamud AY, Patel SC. Intraluminal Carotid Artery Thrombus in COVID-19: Another Danger of Cytokine Storm? *Am J Neuroradiol* 2020;:1–6. doi:10.3174/ajnr.A6674

52 Morelli N, Rota E, Terracciano C, *et al.* The Baffling Case of Ischemic Stroke Disappearance from the Casualty Department in the COVID-19 Era. *Eur Neurol* Published Online First: 2020. doi:10.1159/000507666

53 Morelli N, Rota E, Terracciano C, *et al.* COVID-19-Related Stroke: Barking up the Wrong Tree? Eur. Neurol. 2020;**83**:218–9. doi:10.1159/000509002

54 Nguyen TN, Abdalkader M, Jovin TG, *et al.* Mechanical Thrombectomy in the Era of the COVID-19 Pandemic: Emergency Preparedness for Neuroscience Teams: A Guidance Statement From the Society of Vascular and Interventional Neurology. *Stroke* 2020;:1–6. doi:10.1161/STROKEAHA.120.030100

55 Ntaios G, Michel P, Georgiopoulos G, *et al.* Characteristics and Outcomes in Patients With COVID-19 and Acute Ischemic Stroke. *Stroke* 2020;**51**. doi:10.1161/STROKEAHA.120.031208

56 Ospel JM, Goyal M. Endovascular stroke treatment during the COVID-19 pandemic. *Nat Rev Neurol* Published Online First: 2020. doi:10.1038/s41582-020-0371-1

57 Pasarikovski CR, da Costa L. The Impact of the Covid-19 Pandemic on Stroke Volume. *Can J Neurol Sci / J Can des Sci Neurol* 2020;**41**:1–2. doi:10.1017/cjn.2020.116

58 Paterson RW, Brown RL, Benjamin L, *et al.* The emerging spectrum of COVID-19 neurology: clinical, radiological and laboratory findings. *Brain* 2020;:2–37. doi:10.1093/brain/awaa240

59 Pedicelli A, Valente I, Pilato F, *et al.* Stroke priorities during COVID-19 outbreak: acting both fast and safe. *J Stroke Cerebrovasc Dis* 2020;:104922. doi:10.1016/j.jstrokecerebrovasdis.2020.104922

60 Qureshi AI, Abd-Allah F, Alsenani F, *et al.* Management of acute ischemic stroke in patients with COVID-19 infection: Report of an international panel. *Int J Stroke* 2020;**0**:1–15. doi:10.1177/1747493020923234

61 Qureshi AI, French BR, Siddiq F, *et al.* COVID-19 Screening with Chest CT in Acute Stroke Imaging: A Clinical Decision Model. *J Neuroimaging* 2020;**30**:617–24. doi:10.1111/jon.12746

62 Qureshi AI, Abd-Allah F, Alsenani F, *et al.* Management of acute ischemic stroke in patients with COVID-19 infection: Insights from an international panel. *Am J Emerg Med* Published Online First: 2020. doi:10.1016/j.ajem.2020.05.018

63 Reddy ST, Garg T, Shah C, *et al.* Cerebrovascular Disease in Patients with COVID-19: A Review of the Literature and Case Series. *Case Rep Neurol* 2020;**77030**:199–209. doi:10.1159/000508958

64 Rodríguez-Pardo J, Fuentes B, Alonso de Leciñana M, *et al.* Acute stroke care during the COVID-19 pandemic. Ictus Madrid Program recommendations. *Neurologia* Published Online First: 2020. doi:10.1016/j.nrl.2020.04.008

65 Saggese CE, Del Bianco C, Di Ruzza MR, *et al.* COVID-19 and Stroke: Casual or Causal Role? *Cerebrovasc Dis* 2020;:1–4. doi:10.1159/000509453

66 Salahuddin H, Castonguay AC, Zaidi SF, *et al.* Interventional Stroke Care in the Era of COVID-19. *Front Neurol* 2020;**11**:1–8. doi:10.3389/fneur.2020.00468

67 Sangalli D, Polonia V, Colombo D, *et al.* A single-centre experience of intravenous thrombolysis for stroke in COVID-19 patients. *Neurol Sci* 2020;**41**:2325–9. doi:10.1007/s10072-020-04591-3

68 Shahjouei S, Naderi S, Li J, *et al.* Risk of stroke in hospitalized SARS-CoV-2 infected patients: A multinational study. *EBioMedicine* 2020;**59**:102939. doi:10.1016/j.ebiom.2020.102939

69 Sheth SA, Wu T-C, Sharrief A, *et al.* Early Lessons From World War COVID: Reinventing Our Stroke Systems of Care. *Stroke* 2020;:STROKEAHA120030154. doi:10.1161/STROKEAHA.120.030154

70 Sierra-Hidalgo F, Muñoz-Rivas N, Torres Rubio P, *et al.* Large artery ischemic stroke in severe COVID-19. *J Neurol* 2020;:3–5. doi:10.1007/s00415-020-09967-1

71 Sweid A, Hammoud B, Bekelis K, *et al.* EXPRESS: Brain ischemic and hemorrhagic complications of COVID-19. *Int J Stroke* 2020;:1747493020937189. doi:10.1177/1747493020937189

72 Sylaja PN, Srivastava MVP, Shah S, *et al.* The SARS-CoV-2/COVID-19 pandemic and challenges in stroke care in India. *Ann N Y Acad Sci* 2020;:1–8. doi:10.1111/nyas.14379

73 Tsivgoulis G, Katsanos AH, Ornello R, *et al.* Ischemic Stroke Epidemiology During the COVID-19 Pandemic: Navigating Uncharted Waters With Changing Tides. *Stroke* 2020;:STROKEAHA120030791. doi:10.1161/STROKEAHA.120.030791

74 Valderrama EV, Humbert K, Lord A, *et al.* Severe Acute Respiratory Syndrome Coronavirus 2 Infection and Ischemic Stroke. *Stroke* 2020;:1–4. doi:10.1161/strokeaha.120.030153

75 Varatharaj A, Thomas N, Ellul MA, *et al.* Neurological and neuropsychiatric complications of COVID-19 in 153 patients: a UK-wide surveillance study. *The Lancet Psychiatry* 2020;**2**:1–8. doi:10.1016/s2215-0366(20)30287-x

76 Venketasubramanian N, Hennerici MG. Stroke in COVID-19 and SARS-CoV-1. *Cerebrovasc Dis* 2020;**188770**:1–2. doi:10.1159/000508370

77 Wang X, Chen Y, Li Z, *et al.* Providing uninterrupted care during COVID-19 pandemic: experience from Beijing Tiantan Hospital. *Stroke Vasc Neurol* 2020;:svn-2020-000400. doi:10.1136/svn-2020-000400

78 Yaeger KA, Fifi JT, Yim B, *et al.* Initial Stroke Thrombectomy Experience in New York City during the COVID-19 Pandemic. 2020;:1–4.

79 Yaghi S, Ishida K, Torres J, *et al.* SARS2-CoV-2 and Stroke in a New York Healthcare System. *Stroke* 2020;:STROKEAHA120030335. doi:10.1161/STROKEAHA.120.030335

80 Zhai P, Ding Y, Li Y. The impact of COVID-19 on ischemic stroke. *Diagn Pathol* 2020;**15**:78. doi:10.1186/s13000-020-00994-0

81 Zhao J, Rudd A, Liu R. Challenges and Potential Solutions of Stroke Care During the Coronavirus Disease 2019 (COVID-19) Outbreak. *Stroke* 2020;**364**:2019–20. doi:10.1161/STROKEAHA.120.029701

82 Agarwal S, Scher E, Rossan-Raghunath N, *et al.* Acute stroke care in a New York City comprehensive stroke center during the COVID-19 pandemic. *J Stroke Cerebrovasc Dis* 2020;**29**:105068. doi:10.1016/j.jstrokecerebrovasdis.2020.105068

83 Baracchini C, Pieroni A, Viaro F, *et al.* Acute stroke management pathway during Coronavirus-19 pandemic. *Neurol Sci* Published Online First: 2020. doi:10.1007/s10072-020-04375-9

84 de Havenon A, Ney J, Callaghan B, *et al.* A Rapid Decrease in Stroke, Acute Coronary Syndrome, and Corresponding Interventions at 65 United States Hospitals Following Emergence of COVID-19. *medRxiv Prepr Serv Heal Sci* Published Online First: 2020. doi:10.1101/2020.05.07.20083386

85 Diegoli H, Magalhães PSC, Martins SCO, *et al.* Decrease in Hospital Admissions for Transient Ischemic Attack , Mild , and Moderate Stroke During the COVID-19 Era. 2020;:1–7. doi:10.1161/STROKEAHA.120.030481

86 Esenwa C, Parides MK, Labovitz DL. The effect of COVID-19 on stroke hospitalizations in New York City. *J Stroke Cerebrovasc Dis* 2020;**29**:105114. doi:10.1016/j.jstrokecerebrovasdis.2020.105114

87 Hsiao J, Sayles E, Antzoulatos E, *et al.* Effect of COVID-19 on Emergent Stroke Care: A Regional Experience. *Stroke* 2020;:STROKEAHA120030499. doi:10.1161/STROKEAHA.120.030499

88 Huang JF, Greenway MRF, Nasr DM, *et al.* Telestroke in the Time of COVID-19: The Mayo Clinic Experience. *Mayo Clin Proc* 2020;**95**:1704–8. doi:10.1016/j.mayocp.2020.06.007

89 Jasne AS, Chojecka P, Maran I, *et al.* Stroke Code Presentations, Interventions, and Outcomes Before and During the COVID-19 Pandemic. *Stroke* 2020;**51**:2664–73. doi:10.1161/STR.0000000000000347

90 Kerleroux B, Fabacher T, Bricout N, *et al.* Mechanical Thrombectomy for Acute Ischemic Stroke Amid the COVID-19 Outbreak. *Stroke* Published Online First: 20 May 2020. doi:10.1161/STROKEAHA.120.030373

91 Mehrpour M, Shuaib A, Farahani M, *et al.* EXPRESS: COVID-19 and stroke in Iran; a case series, and effects on stroke admissions. *Int J Stroke* 2020;:1747493020937397. doi:10.1177/1747493020937397

92 Montaner J, Barragán-Prieto A, Pérez-Sánchez S, *et al.* Break in the Stroke Chain of Survival Due to COVID-19. *Stroke* 2020;:STROKEAHA120030106. doi:10.1161/STROKEAHA.120.030106

93 Naccarato M, Scali I, Olivo S, *et al.* Has COVID-19 played an unexpected “stroke” on the chain of survival? *J Neurol Sci* 2020;**414**:116889. doi:10.1016/j.jns.2020.116889

94 Nguyen-Huynh MN, Tang XN, Vinson DR, *et al.* Acute Stroke Presentation, Care, and Outcomes in Community Hospitals in Northern California During the COVID-19 Pandemic. *Stroke* 2020;:1–7. doi:10.1161/strokeaha.120.031099

95 Onteddu SR, Nalleballe K, Sharma R, *et al.* Underutilization of Healthcare for strokes during the

COVID-19 outbreak. *Int J Stroke* 2020;:1747493020934362. doi:10.1177/1747493020934362

96 Paliwal PR, Tan BYQ, Leow AST, *et al.* Impact of the COVID-19 pandemic on hyperacute stroke treatment: experience from a comprehensive stroke centre in Singapore. *J Thromb Thrombolysis* 2020;**2019**. doi:10.1007/s11239-020-02225-1

97 Pandey AS, Daou BJ, Tsai JP, *et al.* Letter: COVID-19 Pandemic—The Bystander Effect on Stroke Care in Michigan. *Neurosurgery* 2020;**0**:2020–2. doi:10.1093/neuros/nyaa252

98 Perry R, Banaras A, Werring DJ, *et al.* What has caused the fall in stroke admissions during the COVID-19 pandemic? *J Neurol* 2020;:1–2. doi:10.1007/s00415-020-10030-2

99 Pop R, Quenardelle V, Hasiu A, *et al.* Impact of the Covid-19 outbreak on acute stroke pathways – Insights from the Alsace region in France. *Eur J Neurol* Published Online First: 2020. doi:10.1111/ene.14316

100 Rudilosso S, Laredo C, Vera V, *et al.* Acute Stroke Care Is at Risk in the Era of COVID-19: Experience at a Comprehensive Stroke Center in Barcelona. *Stroke* 2020;:STROKEAHA120030329. doi:10.1161/STROKEAHA.120.030329

101 Saxhaug Kristoffersen E, Holt Jahr S, Thommessen B, *et al.* Effect of COVID-19 pandemic on stroke admission rates in a Norwegian population. *Acta Neurol Scand* 2020;:0–2. doi:10.1111/ane.13307

102 Schirmer CM, Ringer AJ, Arthur AS, *et al.* Delayed presentation of acute ischemic strokes during the COVID-19 crisis. *J Neurointerv Surg* 2020;:1–4. doi:10.1136/neurintsurg-2020-016299

103 Siegler JE, Heslin ME, Thau L, *et al.* Falling stroke rates during COVID-19 pandemic at a Comprehensive Stroke Center: Cover title: Falling stroke rates during COVID-19. *J Stroke Cerebrovasc Dis* 2020;:104953. doi:10.1016/j.jstrokecerebrovasdis.2020.104953

104 Strasser S, Miskolczi L, Cunha J, *et al.* COVID-19 Impact on Stroke Presentations. *J Stroke Cerebrovasc Dis* 2020;**29**:105077. doi:10.1016/j.jstrokecerebrovasdis.2020.105077

105 Tejada Meza H, Lambea Gil Á, Sancho Saldaña A, *et al.* Ischaemic stroke in the time of coronavirus disease 2019. *Eur J Neurol* 2020;**27**:1788–92. doi:10.1111/ene.14327

106 Tejada Meza H, Lambea Gil Á, Saldaña AS, *et al.* Impact of COVID-19 outbreak on ischemic stroke admissions and in-hospital mortality in North-West Spain. *Int J Stroke* 2020;:174749302093830. doi:10.1177/1747493020938301

107 Teo K-C, Leung WCY, Wong Y-K, *et al.* Delays in Stroke Onset to Hospital Arrival Time During COVID-19. *Stroke* 2020;:STROKEAHA120030105. doi:10.1161/STROKEAHA.120.030105

108 Yang B, Wang T, Chen J, *et al.* Impact of the COVID-19 pandemic on the process and outcome of thrombectomy for acute ischemic stroke. *J Neurointerv Surg* 2020;:1–5. doi:10.1136/neurintsurg-2020-016177

109 Zhao J, Li H, Kung D, *et al.* Impact of the COVID-19 Epidemic on Stroke Care and Potential Solutions. *Stroke* 2020;:STROKEAHA120030225. doi:10.1161/STROKEAHA.120.030225

110 Zini A, Romoli M, Gentile M, *et al.* The stroke mothership model survived during COVID-19 era: an observational single-center study in Emilia-Romagna, Italy. *Neurol Sci* 2020;**41**:3395–9. doi:10.1007/s10072-020-04754-2
